# The carbohydrate utilization regulator Cbr1 coordinates nutrient-specific gene activation with selective carbon catabolite repression in a basidiomycete yeast

**DOI:** 10.1371/journal.pbio.3003983

**Published:** 2026-09-01

**Authors:** Brandon Reyes-Chavez, Joshua D. Kerkaert, Lori B. Huberman

**Affiliations:** 1 Plant Pathology and Plant-Microbe Biology Section, School of Integrative Plant Science, Cornell University, Ithaca, New York, United States of America; 2 Microbiology Department, Cornell University, Ithaca, New York, United States of America; University of Michigan, UNITED STATES OF AMERICA

## Abstract

Cells must sense and respond to nutrients to survive. To efficiently grow in mixed carbon environments, microbes repress genes necessary to utilize carbon sources that require substantial resources to catabolize when a simpler carbon source, such as glucose, is present. This process is known as carbon catabolite repression. Canonically, in fungi, nutrient sensing transcriptional networks are composed of carbon source-specific transcription factors that activate carbon source utilization genes and carbon catabolite repression regulators, which broadly repress all nonpreferred carbon source utilization genes when a preferred carbohydrate is present. In contrast to this model, we identified a transcription factor (Cbr1) in the basidiomycete yeast *Rhodotorula* (*Rhodosporidium*) *toruloides* that specifically inhibits glucose-mediated repression of disaccharide and proline utilization, presenting a mechanism of tailored carbon catabolite repression regulation that combats a negative feedback loop formed when glucose is released during disaccharide utilization. Cbr1 is also required for cellobiose, gentiobiose, carboxylic acid, and fucose utilization. Using transcriptomic and molecular analyses, we demonstrated that catabolism of these carbon sources is not metabolically linked, but genes necessary for their utilization are coactivated by Cbr1 in response to each of the carbon sources. This coactivation suggests *R. toruloides* may encounter these carbon sources together, potentially during complex interactions among microbes in nature. Coregulation of nutrient-specific gene activation and carbon catabolite repression by a transcription factor establishes a previously uncharacterized mechanism for building nutrient sensing transcriptional networks in fungi. Characterizing diverse nutrient sensing regulatory mechanisms is critical for understanding resource acquisition during fungal pathogenesis, where carbon catabolite repression is important for virulence and drug tolerance, and metabolically engineering fungi for green biotechnology.

## Abstract 2

Fungi must sense and prioritize nutrients to compete with microbes and form relationships with plants and animals. This study shows that the regulator Cbr1 coordinates nutrient use and avoids negative feedback that could block access to some food sources.

## Introduction

Cells must sense and respond to nutrients to successfully grow and divide using the resources available. For fungi, nutrient sensing is critical to establish fungal colonies, compete with other microbes, and establish pathogenic and symbiotic relationships with plants and animals. Most work characterizing genetic pathways regulating carbon source utilization in fungi has been performed in the Ascomycete phylum [1,2]. In these fungi, a network of carbon source-specific transcription factors activates genes necessary for utilization of available carbon [3]. However, in a mixed carbon environment, it is not necessarily energetically optimal to simultaneously consume all available carbon sources. Thus, when a more preferred carbon source, such as glucose, is available, a process known as carbon catabolite repression broadly downregulates expression of genes required for utilization of all less preferred carbon sources [1,3–10].

Many devastating human, animal, and plant pathogens fall outside of the Ascomycete phylum [11]. Genetic mechanisms through which these fungi activate genes necessary to utilize nonpreferred carbon sources and repress these genes in the presence of more preferred carbon sources are significantly less well studied. Basidiomycetes diverged from Ascomycetes approximately 400 million years ago and represent over 30% of described fungi [12,13]. Recent studies in Basidiomycete pathogens have demonstrated that understanding the regulation of carbon utilization in this phylum is critical, since accurately regulating carbon metabolism is not only important for virulence but also changes the efficacy of antifungal drug treatments [14–16].

To address this knowledge gap, we investigated genetic mechanisms regulating carbon utilization in the basidiomycete yeast *Rhodotorula toruloides* (previously *Rhodosporidium toruloides*). While *R. toruloides* is in a different phylogenetic class than some of the more established basidiomycete human and plant pathogens, like *Cryptococcus neoformans*, *Puccinia graminis*, or *Ustilago maydis*, *Rhodotorula* species are an emerging cause of opportunistic infections in humans and animals that are frequently resistant to antifungal drugs [17–19]. *Rhodotorula* infections are often associated with central venous catheter use in immunosuppressed individuals, and meningeal, skin, ocular, peritoneal, and prosthetic joint infections can occur in healthy individuals [18,20,21]. Recent studies of Ascomycete yeast species have established these organisms have a range of carbon utilization repertoires, from specialists capable of consuming only a small range of carbohydrates to generalists that have a much broader carbon utilization repertoire [22]. *R. toruloides* is a generalist that can consume most soluble breakdown products of plant biomass [23,24]. *R. toruloides* can also accumulate up to 70% of its biomass as fatty acids in conditions in which carbon is abundant but other nutrients are limiting and produces useful secondary metabolites, such as carotenoids [25–31]. This makes *R. toruloides* an excellent choice for metabolic engineering [32], where understanding the regulation of carbon metabolism is critical [33,34].

Cellulose is the most abundant plant biomass component and is composed of glucose chains linked by β-1,4-glycosidic bonds [35]. *R. toruloides* can utilize the disaccharide cellulose building block, cellobiose [36]. We identified a transcription factor, Cbr1, required for cellobiose utilization. Cbr1 is homologous to the cellulose degradation regulator CLR-2/ClrB in Ascomycetes [37], but the role of Cbr1 is significantly expanded in *R. toruloides*, elucidating alternative mechanisms of building carbon utilization regulatory networks in fungi. Unlike CLR-2/ClrB, Cbr1 is also required for gentiobiose, tricarboxylic acid (TCA) cycle intermediate, and fucose utilization. Cellobiose/gentiobiose, fucose, and TCA cycle intermediates are not catabolized through similar pathways, potentially suggesting *R. toruloides* may inhabit an ecological niche in which these carbon sources are frequently found together, possibly through cooperation with or exploitation of saprophytic filamentous fungi.

Further investigation of Cbr1 demonstrated this transcription factor inhibits carbon catabolite repression specifically of disaccharide and proline utilization genes. This role for Cbr1 contrasts with previously identified regulatory mechanisms of carbon catabolite repression, which regulate utilization of all nonpreferred carbon sources. Taken together, the diverse roles of Cbr1 in *R. toruloides* metabolic regulation present a previously uncharacterized mechanism of carbon catabolite repression and nutrient sensing transcriptional network composition in fungi.

## Results

### Cbr1 is required for wild-type utilization of cellobiose, gentiobiose, TCA cycle intermediates, and fucose

**Fig 1:**
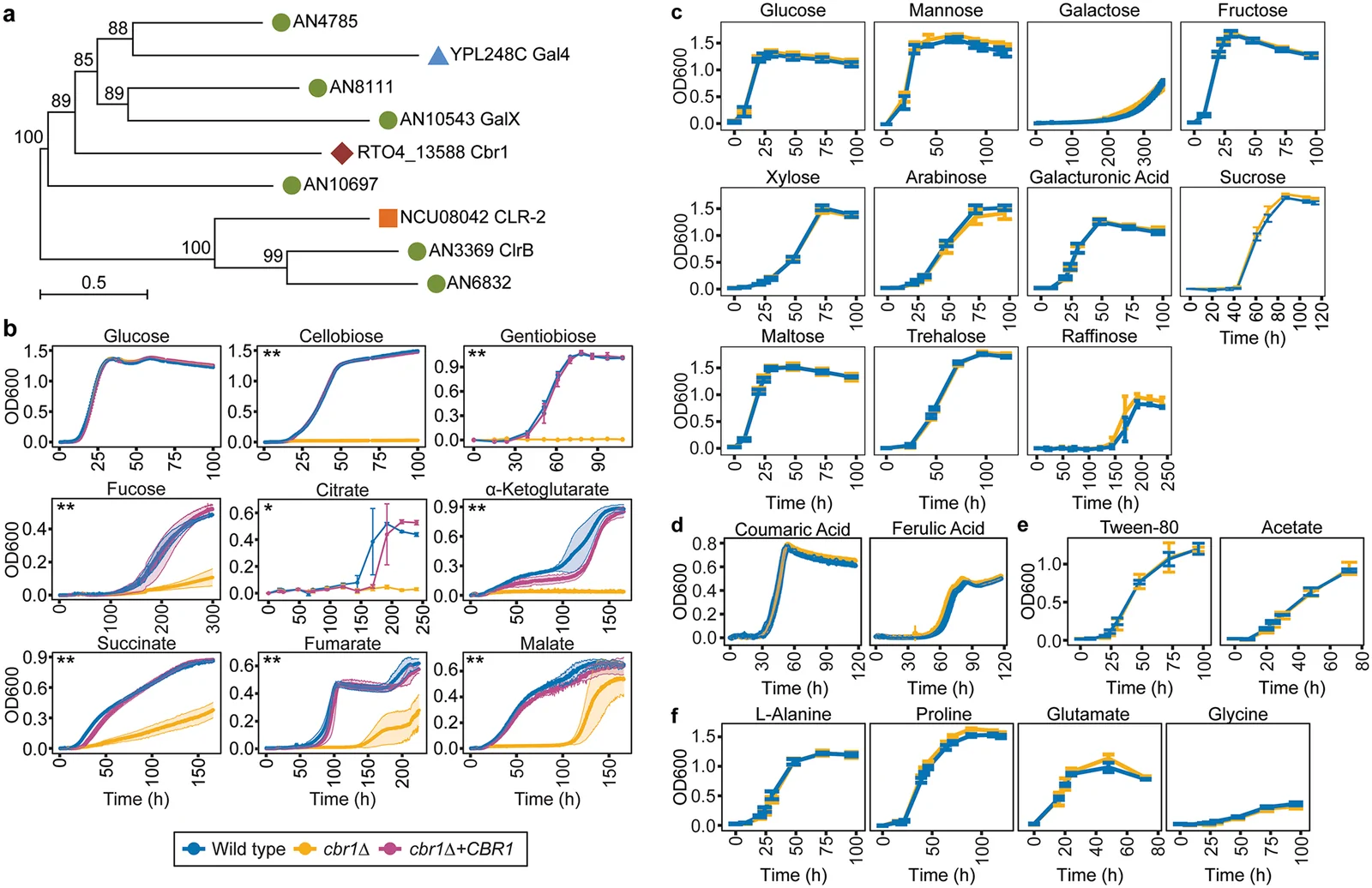
*CBR1* (RTO4_13588) is involved in utilization of cellobiose, gentiobiose, TCA cycle intermediates, and fucose. **(A)** Protein sequences of *N. crassa* CLR-2 and *Aspergillus nidulans* ClrB homologs were used to build a phylogenetic tree using the maximum likelihood method based on the Jones–Taylor–Thornton matrix-based model using FastTree [88]. Numbers at nodes indicate the reliability of each split in the tree as computed using the Shimodaira-Hasegawa test on three alternate topologies around that split with 1,000 resamples. *R. toruloides* proteins are indicated with maroon diamonds. *N. crassa* proteins are indicated with orange squares. *A. nidulans* proteins are indicated with green circles. *S. cerevisiae* proteins are indicated with blue triangles. Scale bar indicates amino acid substitutions per site. The tree file for this phylogenetic tree can be found in S1 Data. **(B–F)** Growth curves (OD600) of the indicated strains grown in yeast nitrogen base (YNB) with the indicated carbon source at 1%, except for gentiobiose at 10 mM, citrate at 8.5 mM, and coumaric and ferulic acid at 0.2%. Cultures were inoculated at an OD600/ml of (**B**) 0.01 and (**C–F**) 0.1 for all conditions except mannose, galactose, sucrose, trehalose, proline, and raffinose, which were inoculated at 0.01. Lines are the average and bars or colored bands are the standard deviation of three biological replicates. The data underlying **B–F** can be found in S2 Data. *p_adj_ < 10^−4^ and **p_adj_ < 10^−10^ for *cbr1*Δ vs. wild type (WT) and *cbr1*Δ vs. *cbr1*Δ + *CBR1* as determined by pairwise comparisons of estimated marginal means with a Holm multiple comparison correction. Exact *p*-values are in S3 Data.

*R. toruloides* can utilize cellobiose, the disaccharide breakdown product of the most abundant plant cell wall polysaccharide, cellulose [36]. Several transcription factors regulate cellulose and cellobiose utilization in Ascomycete filamentous fungi, including CLR-1/ClrA, CLR-2/ClrB, and XlnR [1,37–40]. To investigate genetic mechanisms regulating *R. toruloides* cellobiose utilization, we searched for genes in the *R. toruloides* genome with homology to these transcription factors. No clear homolog of CLR-1/ClrA or XlnR existed in *R. toruloides* (S1 Fig). However, we identified a single homolog of CLR-2/ClrB, RTO4_13588 (Figs 1A and S1).

We hypothesized RTO4_13588 may play a role in regulating cellobiose utilization in *R. toruloides*. To test this hypothesis, we deleted RTO4_13588 and grew the resulting RTO4_13588Δ strain on cellobiose. Growth of cells lacking RTO4_13588 on glucose was indistinguishable from wild-type cells (Fig 1B). However, deletion of RTO4_13588 resulted in a complete lack of growth on cellobiose, which could be complemented by exogenous expression of RTO4_13588 (*p*_adj_ < 10^−10^, pairwise comparison of estimated marginal means) (Fig 1B). The *N. crassa* homolog of Cbr1, CLR-2, also plays a role in mannan utilization leading us to hypothesize that *CBR1* may be required for utilizing mannose, a major mannan building block [41], but *cbr1∆* cells grew as well as wild type on mannose (Fig 1C). The closest homolog to RTO4_13588 in the model yeast *Saccharomyces cerevisiae* is the transcription factor Gal4, which regulates galactose utilization (Fig 1A) [42]. To test whether RTO4_13588 also played a role in regulating galactose utilization in *R. toruloides*, we grew wild-type and RTO4_13588Δ cells with galactose as the sole carbon source. There was no detectable difference in growth between wild-type cells and cells lacking RTO4_13588 on galactose (Fig 1C). Thus, we named RTO4_13588, *CBR1* for cellobiose regulator 1.

*R. toruloides* can grow on a wide variety of soluble carbon sources [23,36]. We asked whether *CBR1* was required for growth on any additional carbon sources. Cells lacking *CBR1* were unable to grow as well as wild-type cells on the β-1,6-glucose-glucose disaccharide gentiobiose and the deoxyhexose sugar fucose, which could be complemented by exogenous expression of *CBR1* (*p*_adj_ < 10^−10^, pairwise comparison of estimated marginal means) (Fig 1B). The growth of *cbr1*Δ cells was indistinguishable from wild-type cells on all other sugars tested (fructose, xylose, arabinose, galacturonic acid, sucrose, maltose, trehalose, and raffinose) and on the lignin phenolic building blocks, coumaric and ferulic acid (Fig 1C and 1D). *CBR1* was not required for utilization of the lipid mimic Tween-80, the fermentation product acetate, or the amino acids L-alanine, proline, glutamate, or glycine, suggesting *cbr1*Δ cells were capable of respiration (Fig 1E and 1F). Thus, *CBR1* does not play a pleiotropic role in nonpreferred carbon source utilization. Given that *cbr1*∆ cells could grow on carbon sources that require the TCA cycle for metabolism, we hypothesized *cbr1*Δ cells would be able to grow on TCA cycle intermediates. To our surprise, *cbr1*Δ cells had significant growth defects on citrate, α-ketoglutarate, succinate, fumarate, or malate relative to wild-type and *cbr1*Δ *+ CBR1* cells (*p*_adj_ < 10^−4^, pairwise comparison of estimated marginal means) (Fig 1B).

The role for Cbr1 in TCA cycle intermediate and fucose utilization could either be an expanded role for Cbr1 in *R. toruloides* relative to CLR-2/ClrB or a previously uncharacterized role for CLR-2/ClrB. To distinguish between these two possibilities, we assessed growth of the model filamentous fungus *Neurospora crassa* wild-type and Δ*clr-2* cells in media containing succinate, α-ketoglutarate, malate, or fucose as the sole carbon source. Wild-type *N. crassa* was only capable of limited growth on TCA cycle intermediates or fucose, and deletion of *clr-2* caused no detectable growth defect (S2 Fig). These data suggest the *CBR1* role in regulating TCA cycle intermediate and fucose utilization is an expanded role for this transcription factor relative to Cbr1 orthologs in Ascomycete filamentous fungi.

### Cbr1 directly or indirectly activates a core regulon of five genes in response to cellobiose, TCA cycle intermediates, and fucose

We had three possible hypotheses for genetic mechanisms through which Cbr1 could either directly or indirectly regulate cellobiose/gentiobiose, TCA cycle intermediate, and fucose utilization: (1) Cbr1 directly or indirectly regulates genes necessary to utilize all three classes of carbon sources in response to any of these carbon sources, (2) Cbr1 directly or indirectly regulates genes necessary to utilize each carbon source only in response to that specific carbon source, or (3) *R. toruloides* utilization of cellobiose/gentiobiose, fucose, and/or TCA cycle intermediates proceeds through atypical metabolic pathways that require the same transporters and/or catabolic enzymes. To distinguish between these three hypotheses, we used transcriptional profiling. We exposed wild-type and *cbr1*Δ cells to yeast nitrogen base (YNB) lacking a carbon source (no carbon) or supplemented with 1% cellobiose, fucose, or succinate for 8 h prior to harvesting the cells for RNA sequencing (RNAseq). The cellobiose, succinate, and no carbon samples were submitted for whole transcript RNAseq, and the fucose samples were submitted for 3′ RNAseq.

Given the limited transcriptional characterization of *R. toruloides* on cellobiose or succinate, we first examined the transcriptional profile of wild-type cells in these conditions relative to carbon starvation. The expression of 1168 genes was at least 4-fold differentially expressed in wild-type cells exposed to cellobiose compared to carbon starvation, with 155 genes upregulated and 1013 genes downregulated. In wild-type cells exposed to succinate, 525 genes were at least 4-fold differentially expressed relative to carbon-starved cells, with 142 genes upregulated and 383 genes downregulated. Intriguingly, over 75% of genes differentially expressed on succinate (405/525) were also differentially expressed on cellobiose, supporting the hypothesis that similar sets of genes are regulated in response to cellobiose and succinate (S3A–S3C, S4A, and S4B Figs; S4 and S5 Data).

Cells lacking *CBR1* also showed substantial transcriptional responses to cellobiose and succinate compared to carbon starvation. This transcriptional response could occur because despite a requirement for *CBR1* in wild-type utilization of cellobiose and succinate, it is not the only transcription factor involved in the cellobiose or succinate response. To test this, we compared the genes differentially expressed on cellobiose and succinate in *cbr1*∆ cells to wild-type cells. Five hundred seventy-nine genes were at least 4-fold differentially expressed in cellobiose compared to carbon starvation. Eighty-one of the 179 genes (45%) upregulated on cellobiose in *cbr1*Δ cells were also upregulated in wild-type cells exposed to cellobiose, and 319 of the 400 genes (80%) downregulated on cellobiose in *cbr1*Δ cells had the same expression pattern in wild-type cells (S3A, S3D, S3F, S4A, and S4C Figs; S4 and S5 Data). Similarly, 742 genes were differentially expressed on succinate compared to carbon starvation in *cbr1*Δ cells. Eighty-six of the 296 genes (29%) upregulated on succinate in *cbr1∆* cells were also upregulated in wild-type cells exposed to succinate, and 256 of 446 genes (57%) downregulated on succinate in *cbr1∆* cells were also downregulated in wild-type cells exposed to succinate (S3B, S3E, S3G, S4B, and S4D Figs; S4 and S5 Data). The significant transcriptional response to cellobiose and succinate combined with the high degree of transcriptional similarity exhibited by *cbr1*Δ and wild-type cells indicates *cbr1*Δ cells still respond to these carbon sources to some extent and suggests other transcription factors may also be involved in the response to cellobiose and succinate.

Although many fungal transcription factor genes are upregulated in the condition in which the transcription factor plays a role, *CBR1* expression was not induced in response to cellobiose or succinate compared to carbon starvation (S5A Fig; S4 and S5 Data). To directly regulate gene expression, Cbr1 must be present in the nucleus. We hypothesized Cbr1 might be transported into the nucleus under conditions where it plays a role. To test this hypothesis, we tagged Cbr1 with green fluorescent protein (mEGFP) and the histone H2A subunit *HTA1* (RTO4_12090) with mRuby2 and exposed these cells to carbon starvation, glucose, cellobiose, or succinate to identify the localization of Cbr1. Cbr1 colocalized with the histone H2A subunit under all four conditions, indicating Cbr1 is expressed and in the nucleus during exposure to glucose, cellobiose, succinate, and carbon starvation (S5B Fig).

**Fig 2:**
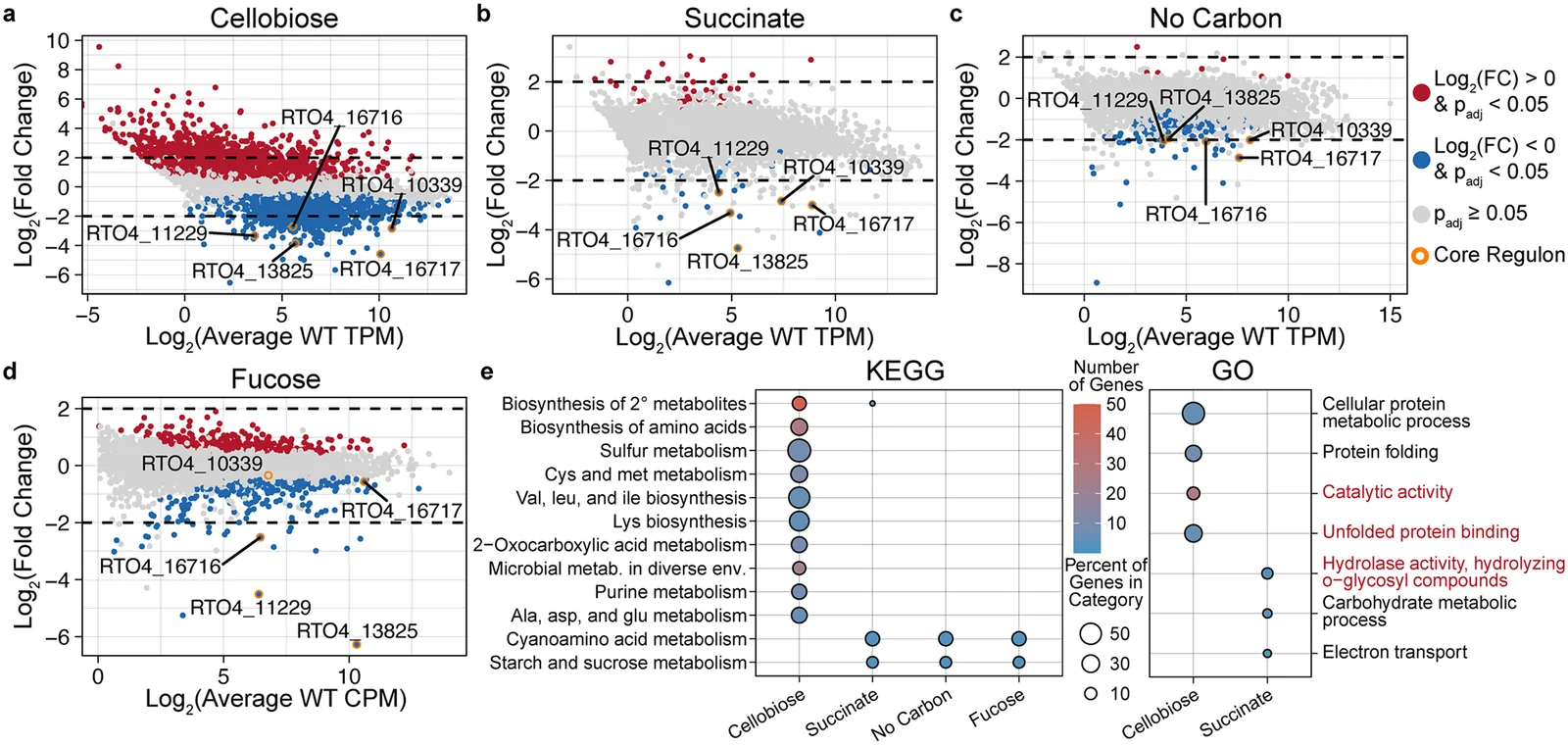
Cbr1 directly or indirectly regulates a core regulon of five genes. **(A–D)** Scatterplots of the log_2_(average expression) of all genes in wild-type (WT) cells vs. the log_2_(fold change) in gene expression in *cbr1*Δ relative to wild-type cells when cells were exposed to (**A**) cellobiose, (**B**) succinate, (**C**) media lacking a carbon source, or (**D**) fucose. Genes with *p*_adj_ < 0.05 are indicated in red or blue if they had higher or lower expression in *cbr1*Δ cells than wild-type cells, respectively. Genes differentially expressed by a least 4-fold in at least three of the four conditions (*CBR1* core regulon) are circled in orange and labeled with their protein IDs. Genes in gray were not significantly differentially expressed in *cbr1*Δ relative to wild-type cells. Black dotted lines indicate 4-fold differential expression. Standard RNAseq (**A–C**) was used to determine gene expression in transcripts per million (TPM) and fold change during exposure to cellobiose, succinate, and carbon starvation. 3′ RNAseq (**D**) was used to determine gene expression in counts per million (CPM) and fold change during exposure to fucose. Standard RNAseq and 3′ RNAseq data cannot be directly compared. All RNAseq experiments had three biological replicates. (**E**) Genes expressed at least 4-fold lower in *cbr1*Δ cells than wild-type cells for each carbon source were tested for Kyoto Encyclopedia of Genes and Genomes (KEGG) [43] (left) and gene ontology (GO) [44,45] (right) functional category enrichment. Comparisons were only displayed if they had at least one statistically significantly enriched category for each analysis. Dot color represents the number of genes differentially expressed belonging to a given category. Dot size represents the percent of genes belonging to a given category that were differentially expressed relative to all genes in that category. The text of GO biological process and molecular function categories are colored black and red, respectively. The expression of *CBR1* was left out of the scatterplots, KEGG, and GO enrichment analyses, since the change in expression of this gene was due to its deletion in the *cbr1*Δ strain.

To gain insight into the mechanism behind the growth defects of cells lacking *CBR1*, we examined genes decreased in expression in *cbr1*Δ relative to wild-type cells. Using a 4-fold differential expression cutoff, 284, 18, 25, and 25 genes were downregulated in *cbr1*Δ compared to wild-type cells on cellobiose, succinate, carbon starvation, and fucose, respectively (Figs 2A–2D and S4E–S4H; S4 and S5 Data). In concordance with our hypothesis that cellobiose, carboxylic acid, and fucose utilization are transcriptionally linked via *CBR1*, genes annotated as part of the Kyoto Encyclopedia of Genes and Genomes (KEGG) [43] 2-oxocarboxylic acid metabolism category were enriched in the *CBR1* regulon on cellobiose (Fig 2E). Carbohydrate metabolism KEGG [43] and gene ontology (GO) [44,45] related categories were enriched on succinate, no carbon, and fucose. Further analysis revealed the carbohydrate-related enrichments primarily consisted of genes predicted to encode β-glucosidases, which cleave cellobiose into two glucose molecules (Fig 2E). Only five genes were differentially expressed by at least 4-fold in at least three of the four conditions: two encoding putative β-glucosidases (RTO4_16716 and RTO4_16717) that are adjacent in the *R. toruloides* genome, two encoding putative major facilitator superfamily transporters (RTO4_10339 and RTO4_13825), and one encoding a putative phosphoglycerate mutase (RTO4_11229) (Figs 2A–2D and S4E–S4H; S4 and S5 Data).

### Cbr1-regulated β-glucosidases are necessary for cellobiose and gentiobiose utilization

**Fig 3:**
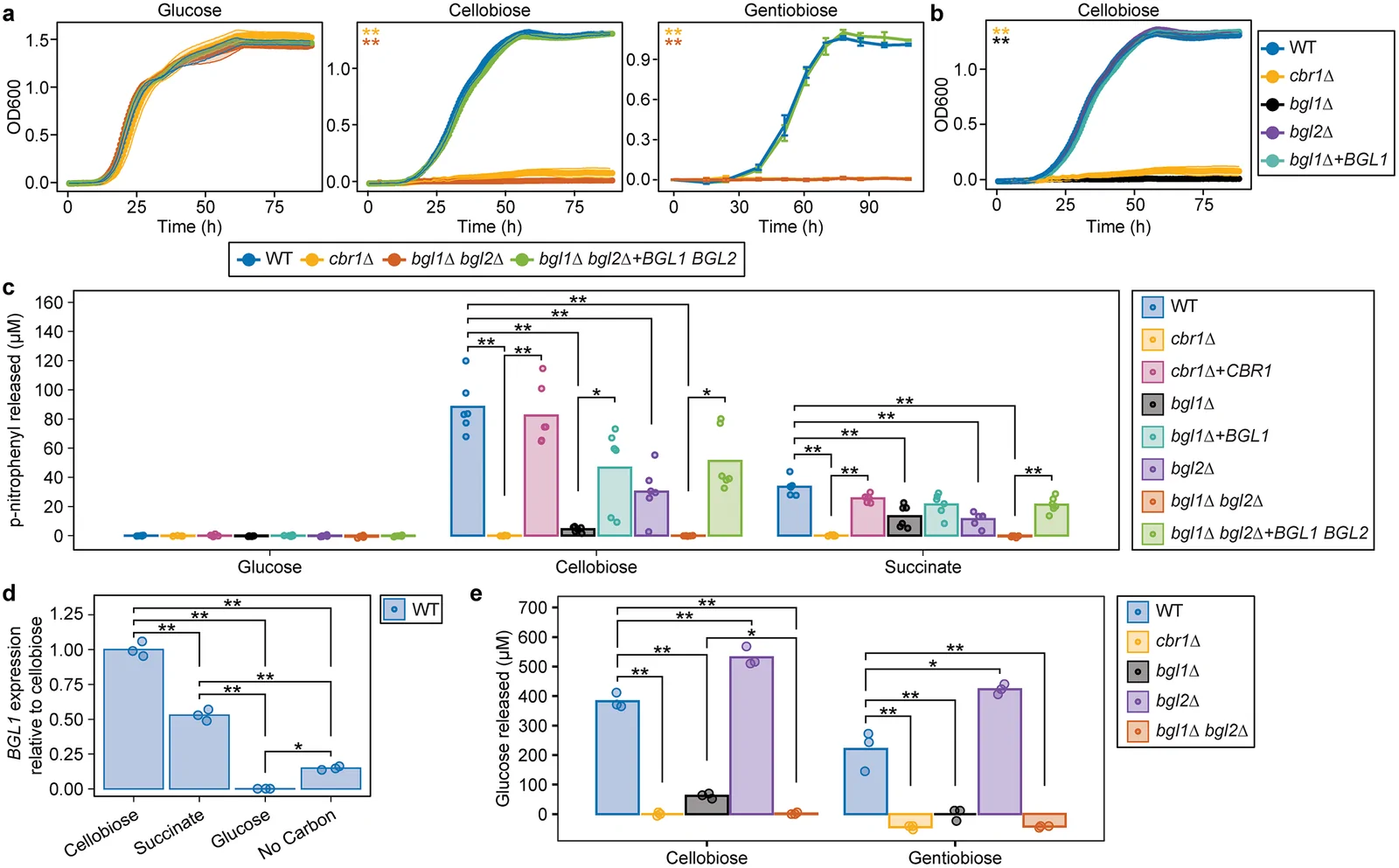
Cbr1-dependent expression of secreted β-glucosidase genes *BGL1* (RTO4_16717) and *BGL2* (RTO4_16716) is necessary for cellobiose and gentiobiose utilization. **(A, B)** Growth (OD600) of the indicated strains in 1% glucose, 1% cellobiose, or 10 mM gentiobiose. (**C**) β-glucosidase activity secreted by the indicated strains into culture supernatants after 20 h growth on the indicated carbon sources. β-glucosidase activity is presented as µM of p-nitrophenyl released from 5 mM p-nitrophenyl-β-D-glucopyranoside. Growth (OD600) at time of supernatant harvest is in S6B Fig. **(D)** *BGL1* expression of WT cells exposed to the indicated carbon source for 4 h relative to *BGL1* expression during exposure to cellobiose for 4 h. **(E)** Glucose released from 1% cellobiose or 1% gentiobiose after incubation with the indicated succinate culture supernatant. Culture supernatants were harvested when a culture reached an OD600 of 0.7. Growth (OD600) at time of supernatant harvest is in S6D Fig. **(A and B)** Lines are the average and bars or colored bands are the standard deviation of three biological replicates. **(C–E)** Bars are the average and dots are the individual data points of (**C**) five (*cbr1∆*) or six (all other strains) biological replicates or (**D–E**) three biological replicates. The data underlying this figure can be found in S2 Data. **(A and B)** Colored stars indicate a significant difference in growth of the respectively colored strain compared to wild-type cells and, when applicable, the respective complement strain. *p_adj_ < 0.05 and **p_adj_ < 10^−4^ as determined by either (**A** and **B**) pairwise comparisons of estimated marginal means with a Holm multiple comparison correction or (**C**–**E**) a one-way ANOVA with a TukeyHSD post-hoc test. Exact *p*-values are in S3 Data.

The *CBR1*-dependent expression of two predicted β-glucosidase genes led us to hypothesize that one or both β-glucosidase genes is essential for cellobiose and/or gentiobiose utilization. An amino acid alignment using LALIGN [46] revealed 72% identity and 90% similarity between the two proteins, suggesting a potential for redundancy, so to test this hypothesis we deleted RTO4_16716 and RTO4_16717, together and individually. RTO4_16716Δ RTO4_16717Δ cells grew like wild-type cells on glucose, citrate, succinate, and fucose, but were unable to grow on cellobiose or gentiobiose (*p*_adj_ < 10^−7^, pairwise comparisons of estimated marginal means) (Figs 3A and S6A). Analysis of the individual deletion mutants revealed that deletion of RTO4_16717 alone eliminated growth on cellobiose and complementation with RTO4_16717 restored the ability to grow on cellobiose (*p*_adj_ < 10^−7^, pairwise comparisons of estimated marginal means) (Fig 3B). Cells lacking RTO4_16716 alone grew as well as wild-type cells on cellobiose (Fig 3B). These data indicate RTO4_16717 encodes the primary β-glucosidase for cellobiose utilization. Thus, we named RTO4_16717 and RTO4_16716, *BGL1* and *BGL2*, respectively, for β-glucosidase 1 and 2.

Both *BGL1* and *BGL2* have predicted secretion signals, leading us to hypothesize cellobiose and gentiobiose cleavage by β-glucosidases occurs extracellularly. To address this hypothesis, we harvested supernatants of wild-type, *cbr1*Δ, and *cbr1*∆ *+ CBR1* cell cultures after 20 h of growth in glucose, cellobiose, or succinate to measure secreted β-glucosidase activity using a p-nitrophenyl-β-D-glucopyranoside colorimetric assay. β-glucosidase activity was detected in the culture supernatant of wild-type cells exposed to cellobiose indicating that β-glucosidases are indeed secreted by *R. toruloides* (Fig 3C). Consistent with our transcriptomics data, and despite the lack of a role for *BGL1* and *BGL2* in carboxylic acid utilization (S6A Fig), β-glucosidase activity was also detectable in the culture supernatant of wild-type cells exposed to succinate. Deletion of *CBR1* eliminated β-glucosidase activity in the supernatant of cellobiose and succinate cultures. This activity was restored in the supernatant of cultures of *cbr1*∆ + *CBR1* cells, indicating the secreted β-glucosidase activity was dependent on *CBR1* (*p*_adj_ < 10^−7^, one-way ANOVA with a TukeyHSD post-hoc test) (Fig 3C).

Because β-glucosidases are not required for glucose utilization, we predicted β-glucosidases would not be secreted into glucose culture supernatants. Indeed, glucose cultures had no detectable β-glucosidase activity, which could either be due to reduced expression of β-glucosidase genes or reduced secretion of β-glucosidase proteins in glucose relative to cellobiose and succinate media (Fig 3C). To distinguish between these possibilities, we performed reverse transcription quantitative PCR (RT-qPCR) on *BGL1* in wild-type cells. Corroborating our RNAseq data, *BGL1* expression was highest on cellobiose, followed by succinate, and then carbon starvation (*p*_adj_ < 0.05, one-way ANOVA with a TukeyHSD post-hoc test) (Fig 3D). During exposure to glucose, *BGL1* expression was nearly undetectable, indicating the lack of β-glucosidase activity in the glucose culture supernatant was due to reduced expression of β-glucosidase genes (Fig 3C and 3D). These data may indicate β-glucosidase expression is regulated by carbon catabolite repression, and that β-glucosidases potentially act as “scout” enzymes during carbon starvation similar to some polysaccharide-degrading enzymes in some filamentous fungi [1,3].

Given that *bgl1*∆ cells cannot grow when cellobiose is the sole carbon source (Fig 3B), we hypothesized Bgl1 was the source of the secreted β-glucosidase activity. To test this hypothesis, we incubated *bgl1*∆ cells in cellobiose, succinate, or glucose for 20 h. While β-glucosidase activity in the cellobiose *bgl1*∆ culture supernatant was 22-fold lower than that of wild-type culture supernatants (*p*_adj_ < 10^−7^, one-way ANOVA with a TukeyHSD post-hoc test), it was detectable, and β-glucosidase activity of succinate *bgl1*∆ cultures was even more pronounced (Figs 3C and S6C). The reduction in β-glucosidase secretion in *bgl1*∆ cellobiose culture supernatants was mitigated in *bgl1*∆ + *BGL1* cells (*p*_adj_ = 0.003, one-way ANOVA with a TukeyHSD post-hoc test) (Fig 3C). The low level of β-glucosidase activity remaining in *bgl1*∆ cells could either result from secretion of Bgl2 or an unidentified protein. To distinguish between these possibilities, we exposed *bgl1*∆ *bgl2*∆ and *bgl1∆ bgl2*∆ + *BGL1 BGL2* cells to cellobiose, succinate, or glucose for 20 h. Cells lacking both *BGL1* and *BGL2* had no detectable β-glucosidase activity, which was rescued by complementation with *BGL1* and *BGL2* (*p*_adj_ < 0.05, one-way ANOVA with a TukeyHSD post-hoc test) (Figs 3C and S6C). These data suggested Bgl2 contributes to β-glucosidase activity in cellobiose and succinate cultures even though *BGL2* was dispensable for growth on cellobiose. Indeed, cells lacking *BGL2* alone had a 2.8-fold reduction of β-glucosidase activity on both cellobiose and succinate relative to wild-type cells, demonstrating that both Bgl1 and Bgl2 are responsible for the secreted β-glucosidase activity of *R. toruloides* (*p*_adj_ < 0.05, one-way ANOVA with a TukeyHSD post-hoc test) (Fig 3C).

To more precisely measure the specific activity of the β-glucosidase enzymes, we measured glucose released from cellobiose and gentiobiose by wild-type, *cbr1*∆, *bgl1*∆, *bgl2*∆, and *bgl1*∆ *bgl2*∆ succinate culture supernatants. We inoculated wild-type, *cbr1*∆, *bgl1*∆, *bgl2*∆, and *bgl1*∆ *bgl2*∆ cells into YNB 1% succinate, grew each culture to an OD600 of 0.7, and then harvested and filter-sterilized culture supernatants. After 48 h of incubation with wild-type culture supernatants, 382 µM glucose was released from 29 mM cellobiose, and 220 µM glucose was released from 29 mM gentiobiose. No glucose was released from cellobiose or gentiobiose by *cbr1*∆ or *bgl1*∆ *bgl2*∆ culture supernatants (*p*_adj_ < 10^−4^, one-way ANOVA with a TukeyHSD post-hoc test) (Fig 3E), indicating that Bgl1 and/or Bgl2 can cleave both β-1,4 and β-1,6 glucose-glucose glycosidic bonds.

To further investigate the roles of Bgl1 and Bgl2 in cellobiose and gentiobiose degradation, we exposed these disaccharides to *bgl1*∆ and *bgl2*∆ succinate culture supernatants. Like we observed using the p-nitrophenyl-β-D-glucopyranoside assay, only a small concentration of glucose (62 µM) was released from cellobiose exposed to *bgl1*∆ succinate culture supernatants (Figs 3E and S6E). Bgl1 appeared even more critical for gentiobiose degradation. No glucose was released from 29 mM gentiobiose by *bgl1*∆ succinate supernatants (Fig 3E). To our surprise, despite reduced p-nitrophenyl release from p-nitrophenyl-β-D-glucopyranoside by *bgl2*∆ relative to wild-type succinate culture supernatants (*p*_adj_ < 10^−4^, one-way ANOVA with a TukeyHSD post-hoc test) (S6E Fig), 39% and 92% more glucose was released from cellobiose and gentiobiose, respectively, by *bgl2*∆ than wild-type culture supernatants (*p*_adj_ < 0.05, one-way ANOVA with a TukeyHSD post-hoc test) (Fig 3E). This increased glucose release may suggest that enzymes that can cleave cellobiose and gentiobiose glycosidic bonds but not the bond of the sugar analog, p-nitrophenyl-β-D-glucopyranoside, were more readily secreted or that the genes encoding these enzymes were activated in a compensatory fashion in cells lacking *BGL2*.

Although we did not detect a role for *CBR1* in regulating utilization of any other disaccharides, we asked whether Bgl1 and Bgl2 activity was specific to β linkages between glucose molecules or whether they could cleave other sugar bonds. We exposed trehalose (α-1,1-glucose-glucose disaccharide), maltose (α-1,4-glucose-glucose disaccharide), lactose (β-1,4-glucose-galactose disaccharide), and sucrose (α-1,2-glucose-fructose disaccharide) to wild-type, *cbr1*∆, and *bgl1*∆ *bgl2*∆ succinate culture supernatants for 48 h. No glucose was released from maltose, lactose, or sucrose by wild-type, *cbr1*∆, or *bgl1*∆ *bgl2*∆ culture supernatants (S6F Fig). Intriguingly, 632 µM glucose was released from 29 mM trehalose (S6F Fig). This glucose release was not dependent on Bgl1 and Bgl2 because glucose released from *bgl1*∆ *bgl2*∆ culture supernatants was indistinguishable from wild-type culture supernatants. However, exposure of trehalose to *cbr1*∆ succinate culture supernatant resulted in a 40% reduction in glucose release compared to exposure to wild-type culture supernatant (*p*_adj_ < 0.05, one-way ANOVA with a TukeyHSD post-hoc test) (S6F Fig). Thus, Bgl1 and Bgl2 activity is specific to β-glucose-glucose glycosidic bonds, but other enzymes with trehalase activity are secreted by *R. toruloides* during exposure to succinate in a manner that is only partially dependent on *CBR1*.

**Fig 4:**
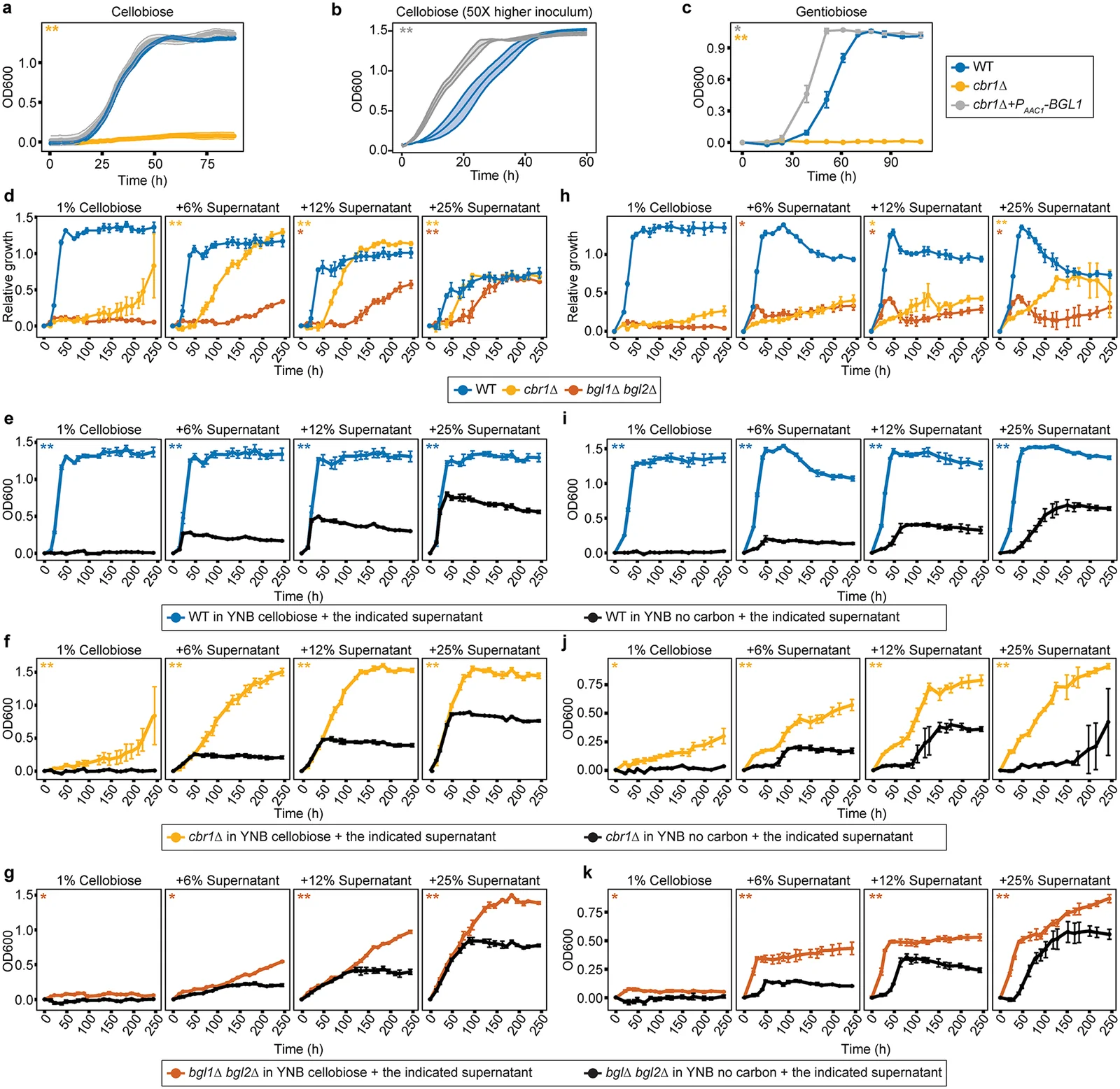
Genes encoding secreted β-glucosidases are the only Cbr1-regulated genes necessary for cellobiose and gentiobiose utilization. **(A–C)** Growth (OD600) of the indicated strains in (**A–B**) 1% cellobiose or (**C**) 10 mM gentiobiose. **(D and H)** Normalized growth (OD600) of the indicated strains in 1% cellobiose supplemented with the indicated percentage of spent supernatant from wild-type cells grown in (**D**) 1% cellobiose or (**H**) 1% succinate. To account for growth on nutrients remaining in the spent supernatant, growth was normalized by subtracting the growth of each replicate on media lacking a carbon source supplemented with the same concentration of spent supernatant. **(E–K)** Growth curves (OD600) of the indicated strains grown in YNB 1% cellobiose and YNB no carbon supplemented with the indicated concentration of culture supernatant from wild-type cells grown on (**E–G**) cellobiose or (**I–K**) succinate without normalization. β-glucosidase activity present in spent supernatants is in S7C Fig. Lines are the average and bars or colored bands are the standard deviation of three biological replicates. The data underlying this figure can be found in S2 Data. **(A–C)** Colored stars indicate a significant difference in growth of the respectively colored strain compared to wild-type cells. **(D and H)** Colored stars indicate a significant difference in growth of the respectively colored strain compared to growth of that strain without supernatant supplementation. **(E–K)** Colored stars indicate a significant difference in growth of the respectively colored strain in YNB cellobiose + the indicated concentration of supernatant compared to YNB no carbon + the indicated concentration of supernatant. *p_adj_ < 0.05 and **p_adj_ < 10^−7^ as determined by pairwise comparisons of estimated marginal means with a Holm multiple comparison correction. Exact p-values are in S3 Data.

It was possible either that β-glucosidase genes were the only Cbr1-regulated genes necessary for cellobiose and gentiobiose utilization or that other genes were also required. To distinguish between these two possibilities, we expressed *BGL1* under the constitutive *AAC1* (RTO4_12704) promoter [47] in a *cbr1*Δ background (*cbr1∆* + *P*_*AAC1*_*-BGL1*). Constitutive expression of *BGL1* in *cbr1*Δ cells resulted in wild-type levels of growth on cellobiose when cells were inoculated at very low densities and a growth advantage on cellobiose relative to wild-type cells at higher starting densities (*p*_adj_ < 10^−7^, pairwise comparisons of estimated marginal means [Fig 4A and 4B]; *p*_adj_ = 0.008, Welch’s two-sample *t* test [S7A Fig]) (Figs 4A, 4B, and S7A). Correspondingly, *cbr1*∆ + *P*_*AAC1*_*-BGL1* cellobiose culture supernatants had 3.2-fold higher β-glucosidase activity than wild-type culture supernatants (*p*_adj_ = 0.01, Welch’s two-sample *t* test) (S7B Fig). Similarly, *cbr1*∆ + *P*_*AAC1*_*-BGL1* cells grew significantly faster than wild-type cells on gentiobiose (*p*_adj_ = 0.003, pairwise comparisons of estimated marginal means) (Fig 4C).

If β-glucosidase activity is the only Cbr1-regulated requirement for cellobiose utilization, we speculated *cbr1*Δ and *bgl1*Δ *bgl2*Δ cell growth on cellobiose could be rescued by exogenously supplied β-glucosidases. To test this hypothesis, we inoculated wild-type, *cbr1*Δ, and *bgl1*Δ *bgl2*Δ cells into cellobiose supplemented with filtered, spent supernatant from wild-type cells grown on cellobiose, succinate, or glucose. Supplementation with spent cellobiose or succinate supernatant enabled significantly improved growth of *cbr1*Δ and *bgl1*Δ *bgl2*Δ cells when added at concentrations higher than 6% or 12%, respectively (*p*_adj_ < 0.05, pairwise comparisons of estimated marginal means) (Fig 4D and 4H). This growth was not solely due to growth on residual carbon or secreted waste products in the spent supernatant, since the growth of these strains on media lacking a carbon source with the same concentration of spent supernatant was significantly less (*p*_adj_ < 0.05, pairwise comparisons of estimated marginal means) (Fig 4E–4G and 4I–4K). Additionally, supplementing cellobiose media with up to 12% or 25% spent glucose supernatant did not enable growth of *cbr1*∆ or *bgl1*∆ *bgl2*∆ cells, respectively (S7D–S7G Fig). Our data demonstrate genes encoding secreted β-glucosidases, activated in response to cellobiose and TCA cycle intermediates, are the only Cbr1-regulated requirement for cellobiose and gentiobiose utilization.

### CBR1-mediated transcriptional response to cellobiose is dependent on extracellular β-glucosidases

Extracellular degradation of cellobiose by *R. toruloides* provoked the question of whether cellobiose activates the *CBR1*-dependent transcriptional response to cellobiose or if the activating signal is a cellobiose catabolic derivative. We differentiated between these possibilities in two ways.

We first compared the transcriptional profile of *bgl1*Δ *bgl2*Δ cells exposed to cellobiose, which have no extracellular β-glucosidase activity but contain wild-type copies of *CBR1*, to that of wild-type and *cbr1*Δ cells using 3′ RNAseq. Expression of *BGL1* was indistinguishable at 4 h and 8 h, so we performed 3′ RNAseq at 4 h to limit the time *cbr1*∆ cells starved on cellobiose (S8A Fig). Although 3′ RNAseq and mRNAseq are not directly comparable due to differences in the library preparation protocol, even given that we performed mRNAseq on cellobiose after an 8 h incubation and 3′ RNAseq on cellobiose after a 4 h incubation, gene expression correlated well between the two RNAseq methods (S8B and S8C Figs). The 3′ RNAseq data also allowed us to improve annotation of the *R. toruloides* 3′ untranslated regions (UTRs) (S6 Data).

**Fig 5:**
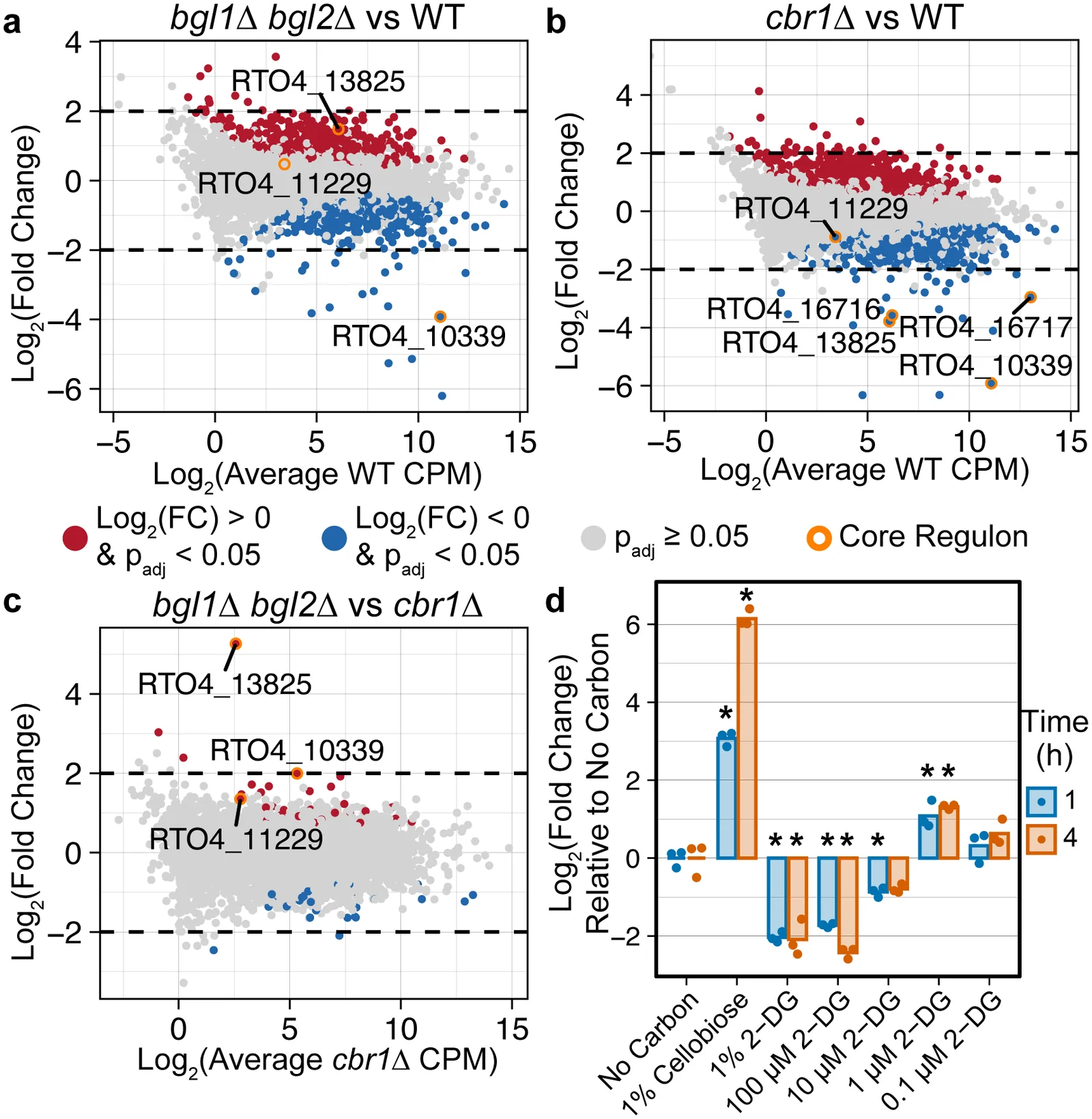
*CBR1*-mediated transcriptional response to cellobiose is dependent on secreted β-glucosidases. **(A–C)** Scatterplots of the log_2_(average expression) of all genes in (**A, B**) wild-type (WT) or (**C**) *cbr1*Δ cells vs. the log_2_(fold change) in gene expression (CPM) for the indicated comparison when cells were exposed to cellobiose, as measured by 3′ RNAseq. Expression of 3′ RNAseq data was normalized using CPM. Genes with *p*_adj_ < 0.05 are indicated in red or blue if they had higher or lower expression, respectively, in (**A** and **C**) *bgl1*Δ *bgl2*Δ cells or (**B**) *cbr1*Δ cells. The genes in the *CBR1* core regulon are circled in orange and labeled with their respective protein IDs. The expression of *CBR1*, *BGL1,* and *BGL2* were left out of the scatterplots involving their respective deletion strains. All RNAseq experiments had three biological replicates. **(D)** *BGL1* expression in wild-type cells exposed to carbon starvation for 1 h followed by exposure to continued carbon starvation or the indicated concentrations of cellobiose or 2-deoxyglucose (2-DG) as the sole carbon source for 1 h or 4 **h.** Expression values were normalized to the average expression of *BGL1* on no carbon for each time point and log_2_ transformed. Bars are the average and dots are the values of three biological replicates. The data underlying **D** can be found in S2 Data. *p_adj_ < 0.05 relative to the no carbon condition for the respective time point, as determined by two-way ANOVA with a TukeyHSD post-hoc test. Exact *p*-values are in S3 Data.

The transcriptional profiles of *bgl1*Δ *bgl2*Δ cells were more similar to *cbr1*Δ than wild-type cells (Figs 5A–5C, and S9; S7 and S8 Data). Thirty-five genes were at least 4-fold differentially expressed in *bgl1*Δ *bgl2*Δ relative to wild-type cells (12 upregulated and 23 downregulated) (Figs 5A and S9B; S7 and S8 Data). Twenty-three of these genes were also at least 4-fold differentially expressed in *cbr1*Δ compared to wild-type cells (Figs 5B and S9C; S7 and S8 Data). In contrast, only five genes were at least 4-fold differentially expressed in *bgl1*Δ *bgl2*Δ relative to *cbr1*Δ cells, which included a 38-fold upregulation of the *CBR1*-regulated predicted transporter gene RTO4_13825 (Figs 5C and S9D; S7 and S8 Data). The expression pattern of RTO4_13825, raised the question of whether any other genes in the core Cbr1 regulon were involved in cellobiose sensing or utilization. Cells lacking RTO4_13825, RTO4_10339, or RTO4_11229 all grew as well as wild type on cellobiose (S10 Fig). These data indicate the signal for the *CBR1*-dependent cellobiose response requires secreted β-glucosidases, suggesting cellobiose is likely not the signaling molecule for Cbr1 activation.

β-glucosidases cleave cellobiose into two glucose molecules. Thus, a second way we answered the question of whether cellobiose or a cellobiose catabolic derivative is the activation signal was to determine whether low glucose concentrations activated Cbr1-regulated gene expression. To mitigate changes in glucose concentration due to metabolism, we used the non-metabolizable glucose analog 2-deoxyglucose. We exposed wild-type cells to 10-fold dilutions of 2-deoxyglucose (100 μM to 0.1 μM), 1% 2-deoxyglucose, 1% cellobiose, or carbon starvation for 1 h and 4 h and measured *BGL1* transcription via RT-qPCR. As expected, cells exposed to cellobiose expressed *BGL1* at high levels, and *BGL1* expression was repressed during exposure to 1% 2-deoxyglucose (*p*_adj_ < 0.05, two-way ANOVA with a TukeyHSD post-hoc test) (Fig 5D). Exposure to 1 μM 2-deoxyglucose resulted in a 2- to 3-fold increase in *BGL1* expression relative to carbon starvation at both timepoints (*p*_adj_ < 0.05, two-way ANOVA with a TukeyHSD post-hoc test) (Fig 5D). This *BGL1* activation did not rise to the same magnitude as exposure to cellobiose itself, perhaps due to differences in glucose concentration, the rate glucose enters the cell, or a lack of resources due to starvation on 2-deoxyglucose. However, increased *BGL1* expression on 1 μM 2-deoxyglucose suggests a low concentration of glucose, a catabolic cellobiose derivative, activates β-glucosidase expression.

### A Cbr1-regulated transporter gene is necessary for citrate utilization

**Fig 6:**
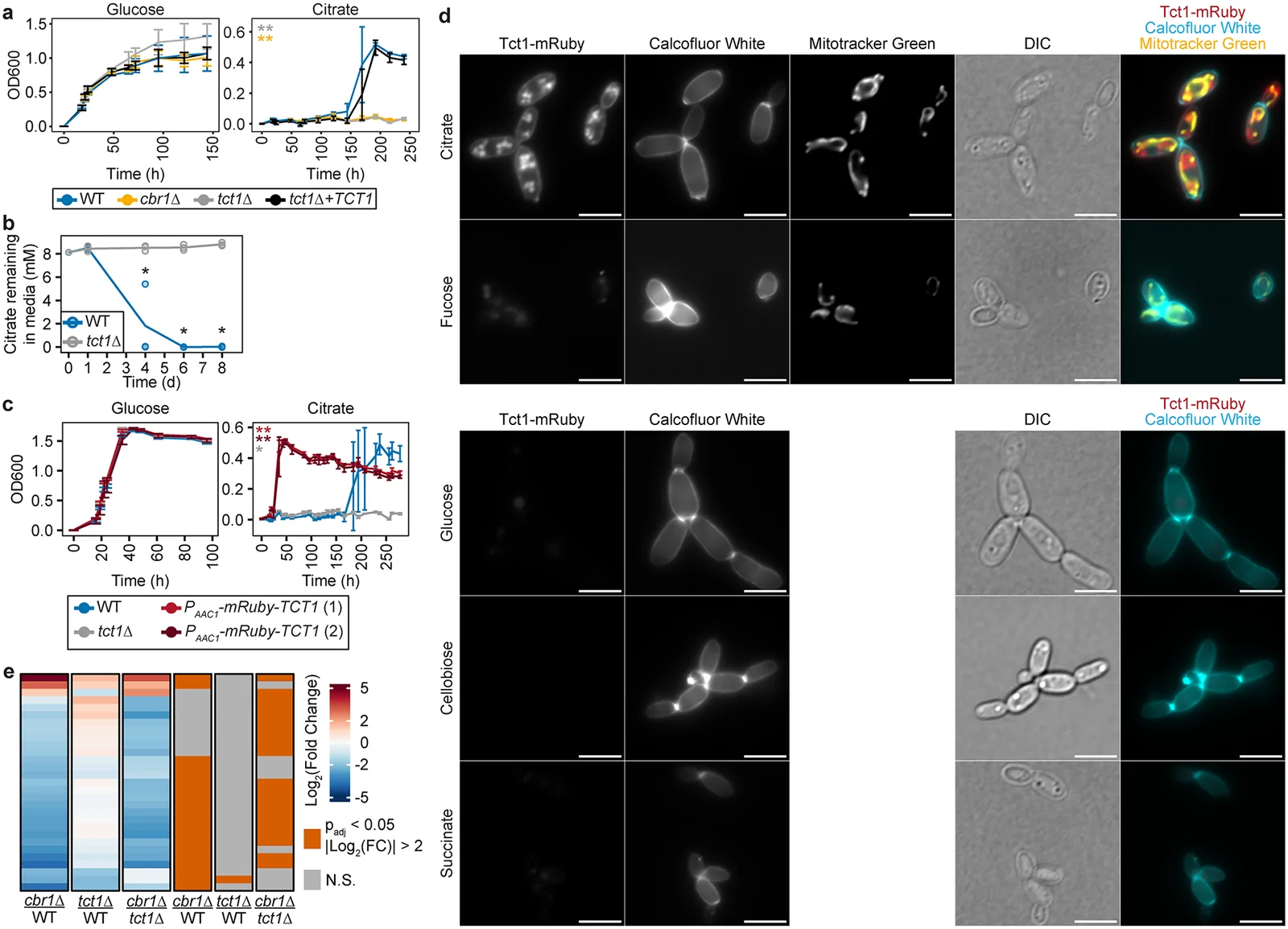
*TCT1* (RTO4_13825) is required for citrate utilization. **(A)** Growth (OD600) of the indicated strains in YNB 1% glucose or 8.5 mM citrate. **(B)** Citrate remaining in YNB 8.5 mM citrate inoculated with the indicated strain at the indicated amount of time post-inoculation. **(C)** Growth (OD600) of the indicated strains in YNB 1% glucose or 8.5 mM citrate. **(D)** Cells with Tct1 tagged with mRuby2 were grown to exponential phase in YNB with the indicated carbon source. Prior to imaging, cells were stained with calcofluor white to stain cell peripheries and, where indicated, mitotracker green to stain mitochondria. Fluorescent microscopy was performed using a Nikon Eclipse Ti2-E inverted microscope equipped with a prime BSI express CMOS camera using a CFI Plan Apochromat 100× oil objective. The scale bar is 5 µm. Images are representative of three biological replicates. **(E)** Heatmap of the log_2_(fold change) in the expression of genes at least 4-fold differentially expressed in one or more of the indicated comparisons when cells were exposed to citrate as the sole carbon source, as measured by 3′ RNAseq. Expression of 3′ RNAseq data was normalized using CPM. Orange bars to the right of the heatmap indicate significance using the criteria of *p*_adj_ < 0.05 and at least 4-fold change in expression, while gray bars indicate genes that do not meet those criteria in the indicated comparison (N.S., not significant). The expression of *CBR1* and *TCT1* were left out of the heatmaps since changes in expression were due to their respective deletions in the *cbr1*Δ and *tct1*Δ strains. All RNAseq experiments had three biological replicates. **(A and C)** Lines are the averages and bars are the standard deviation of three biological replicates. Colored stars indicate a significant difference in growth of the respectively colored strain compared to wild-type cells and, when applicable, the respective complement strain. **(B)** Lines are the averages and dots are the data points of three biological replicates. Stars indicate a significant difference in citrate remaining in the media between wild-type and *tct1∆* cultures at the indicated time point. The data underlying **A–C** can be found in S2 Data. *p_adj_ < 10^−4^ and **p_adj_ < 10^−7^ as determined by (**A** and **C**) pairwise comparisons of estimated marginal means with a Holm multiple comparison correction or (**B**) a two-way ANOVA with a TukeyHSD post-hoc test. Exact *p*-values are in S3 Data.

Two genes encoding predicted major facilitator superfamily transporters (RTO4_13825 and RTO4_10339) and a gene encoding a predicted phosphoglycerate mutase (RTO4_11229) were also members of the *CBR1* core regulon. We hypothesized these genes may be necessary for growth on TCA cycle intermediates and/or fucose. To test this hypothesis, we grew RTO4_13825Δ, RTO4_10339Δ, and RTO4_11229Δ cells on media containing glucose, fucose, citrate, succinate, α-ketoglutarate, malate, or fumarate. The growth of RTO4_10339∆ and RTO4_11229∆ cells was indistinguishable from wild-type cells under all conditions tested (S10 Fig). Cells lacking RTO4_13825 were unable to grow on citrate but grew as well as wild-type cells on all other carbon sources tested (*p*_adj_ < 10^−7^, pairwise comparisons of estimated marginal means) (Figs 6A and S10A).

To confirm that the growth defect of RTO4_13825∆ cells was due to an inability to import citrate rather than an inability to metabolize citrate, we measured citrate uptake in wild-type and RTO4_13825∆ cells. To limit differences in citrate uptake due to citrate metabolism in wild-type but not RTO4_13825∆ cells, we inoculated both wild-type and RTO4_13825∆ cells into YNB citrate at an OD600 of 2, and wild-type cells only reached an average OD600 of 2.6 over the eight-day incubation (S11A Fig). Despite this, all citrate was taken up by wild-type cells after six days. In contrast, no citrate was taken up by RTO4_13825∆ cells even after eight days (*p*_adj_ < 10^−4^, two-way ANOVA with a TukeyHSD post-hoc test) (Fig 6B). To further confirm this difference was due to citrate uptake, rather than growth on citrate, we also inoculated wild-type and RTO4_13825∆ cells into a citrate-phosphate buffer, which lacked nitrogen, sulfur, and trace elements necessary for substantial *R. toruloides* growth, and incubated them for 11 days. Wild-type cells imported two-thirds of the available citrate, while RTO4_13825∆ cells did not take up any citrate after 11 days (*p*_adj_ = 0.0012, two-way ANOVA with a TukeyHSD post-hoc test) (S11B Fig). Thus, we named RTO4_13825 *TCT1* for tricarboxylic acid transporter 1.

If Tct1 imports citrate into the cell, it should be present at the plasma membrane. To test this, we N-terminally tagged Tct1 with mRuby2 and expressed it under a constitutive promoter [47] (*P*_*AAC1*_*-mRuby2-TCT1 tct1*∆). The growth of cells with constitutively expressed *TCT1* was significantly faster than that of wild-type cells on citrate but indistinguishable from wild-type growth on all other carbon sources (*p*_adj_ < 10^−7^, pairwise comparisons of estimated marginal means) (Figs 6C and S11C). During growth on citrate, Tct1 was primarily localized to the plasma membrane and intracellular puncta (Fig 6D). Citrate transporters can also localize to the mitochondria [48,49], but the Tct1-mRuby intracellular puncta did not co-localize with mitochondria (Fig 6D). To our surprise, despite constitutive *TCT1* expression, limited fluorescent signal was visible in *P*_*AAC1*_*-mRuby2-TCT1 tct1*∆ cells grown on glucose, cellobiose, or succinate (Fig 6D). *TCT1* mRNA is highly expressed during fucose exposure (Fig 2D), and Tct1-mRuby fluorescent signal in intracellular puncta was visible during exposure to fucose. However, we did not detect Tct1 localized to the plasma membrane on fucose (Fig 6D). Thus, Tct1 citrate specificity appears to be regulated, at least in part, by protein localization.

To gain further insight into the role of *TCT1*, we conducted RNAseq on wild-type, *cbr1*Δ, and *tct1*Δ cells exposed to citrate for 24 h. Despite the significant growth defect demonstrated by *cbr1*∆ cells on citrate and similar to the number of genes regulated by Cbr1 during exposure to succinate, only 20 genes were at least 4-fold differentially expressed in *cbr1*Δ relative to wild-type cells (Figs 6E and S12A; S9 and S10 Data). Surprisingly, these 20 genes did not include *TCT1*, perhaps due to the observed regulation at the protein level (Fig 6D), or genes predicted to play a role in citrate metabolism. Of the other genes in the *CBR1* core regulon, *BGL1* and *BGL2* were downregulated by 6.7- and 3.2-fold, respectively, in *cbr1*Δ relative to wild-type cells, but RTO4_10339 and RTO4_11229 were not differentially expressed (Figs 6E and S12A; S9 and S10 Data). Only one gene, RTO4_15140, had decreased expression in *tct1*Δ cells relative to wild-type cells. RTO4_15140 was also downregulated in *cbr1*Δ relative to wild-type cells (Figs 6E and S12; S9 and S10 Data). RTO4_15140 is predicted to encode a P-type ATPase transduction domain A containing protein with a single transmembrane domain, and any potential role in citrate utilization is unclear. The similarity of *tct1*Δ and wild-type cell transcriptional profiles may suggest *tct1*Δ cells can sense citrate, despite not being able to grow.

### Cbr1 directly or indirectly inhibits carbon catabolite repression during disaccharide and proline utilization

**Fig 7:**
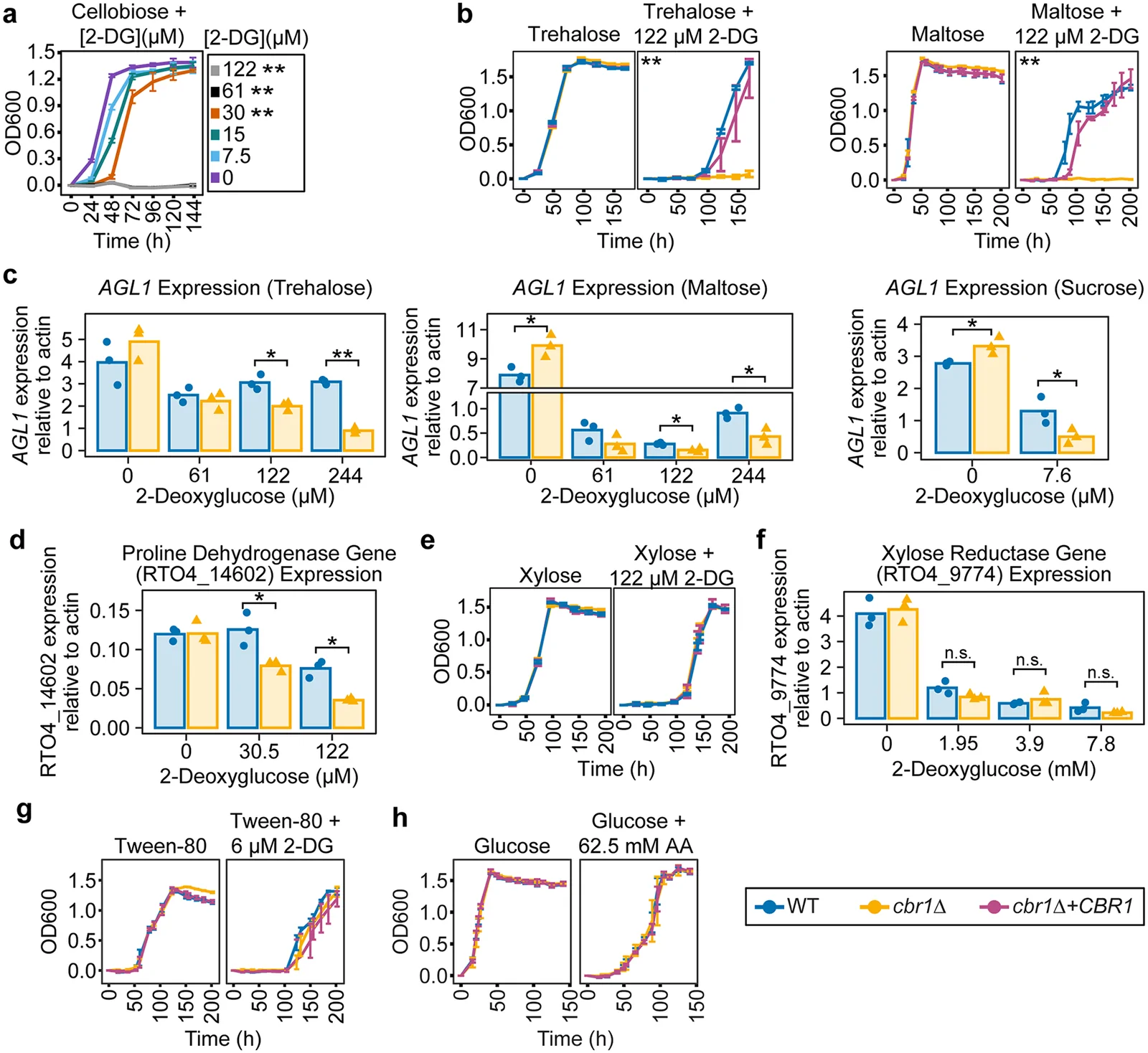
Cbr1 directly or indirectly inhibits carbon catabolite repression during disaccharide and proline utilization. **(A)** Growth (OD600) of wild-type (WT) cells in 1% cellobiose supplemented with the indicated concentration of 2-deoxyglucose (2-DG). (**B, E, G,** and **H**) Growth (OD600) of the indicated strains on the indicated carbon sources with the indicated concentration of 2-DG or allyl alcohol (AA). (**C, D,** and **F**) Expression in wild-type and *cbr1*∆ cells relative to *ACT1* (RTO4_14107) of **(C)** *AGL1* during exposure to trehalose, maltose, or sucrose with the indicated concentration of 2-deoxyglucose, (**D**) the predicted proline dehydrogenase gene RTO4_14602 during exposure to proline with the indicated concentration of 2-deoxyglucose, or (**F**) the xylose reductase gene RTO4_9774 during exposure to xylose with the indicated concentration of 2-deoxyglucose. (**A, B, E, G,** and **H**) Lines are the average and bars are the standard deviation of 3 biological replicates. (**C, D,** and **F**) Bars are the average and dots are individual replicates of 3 biological replicates. The data underlying this figure can be found in S2 Data. *p_adj_ < 0.05 and **p_adj_ < 10^−4^ for (**A**) 1% cellobiose supplemented with the indicated 2-DG concentration vs. 1% cellobiose or (**B**) *cbr1*∆ vs. WT and *cbr1*∆ vs. *cbr1*∆ + *CBR1* as determined by pairwise comparisons of estimated marginal means with a Holm multiple comparison correction and (**C, D,** and **F**) *cbr1*∆ vs. WT as determined by a Welch’s *t* test with a Benjamini–Hochberg multiple hypothesis correction (n.s., not significant). Exact *p*-values are in S3 Data.

Glucose is a highly preferred carbon source in most fungi. Extracellular β-glucosidases cleave cellobiose into two glucose molecules, which we hypothesized would repress cellobiose utilization genes via carbon catabolite repression [1,3,4]. *BGL1* expression is almost undetectable when wild-type cells are exposed to 1% glucose or at least 100 µM 2-deoxyglucose (Figs 3D and 5D). Thus, we hypothesized wild-type cell growth on cellobiose would be inhibited by the non-hydrolyzable glucose analog 2-deoxyglucose. Indeed, 61 µM 2-deoxyglucose fully inhibited *R. toruloides* growth on 1% cellobiose, indicating glucose is preferred over cellobiose by *R. toruloides* (*p*_adj_ < 10^−4^, pairwise comparisons of estimated marginal means) (Fig 7A).

This carbon preference represents a biological challenge during utilization of substrates in which glucose is released during catabolism; a negative feedback loop could repress substrate utilization genes in response to the released glucose, slowing growth on the carbon source. We hypothesized *CBR1* may inhibit carbon catabolite repression to bypass this negative feedback loop. To test this hypothesis, we grew wild-type, *cbr1*Δ, and *cbr1*Δ + *CBR1* cells on a nonpreferred carbon source and 2-deoxyglucose. Cells lacking *CBR1* were significantly more sensitive to 2-deoxyglucose than wild-type or *cbr1*Δ + *CBR1* cells when the glucose-glucose disaccharides maltose or trehalose were the carbon source, suggesting Cbr1 is either a direct or indirect negative regulator of carbon catabolite repression (Figs 7B, S13A, and S13B).

Changes in 2-deoxyglucose sensitivity could be due to differences in gene expression between wild-type and *cbr1*∆ cells or due to post-transcriptional regulation. To distinguish between these possibilities, we first had to identify genes that were activated in response to maltose and trehalose. We searched the *R. toruloides* proteome for proteins with homology to the *S. cerevisiae* maltases Mal12 and Mal32, which can cleave both maltose and sucrose [50–52]. The best hit was RTO4_9135, which was 42.2% identical and 58.3% similar to both Mal12 and Mal32 by an EMBOSS NEEDLE alignment [53]. RTO4_9135 expression was upregulated on maltose, trehalose, sucrose (at 24 h), and xylose relative to all other carbon sources (*p*_adj_ < 0.05, two-way ANOVA with a TukeyHSD post-hoc test) (S14A Fig). RTO4_9135∆ cells were unable to grow on maltose and had a significant growth defect on sucrose but could grow as well as wild type on glucose, trehalose, and cellobiose (*p*_adj_ = 0, pairwise comparisons of estimated marginal means) (S14B Fig). Thus, we named RTO4_9135 *AGL1* for α-glucosidase 1.

*AGL1* expression in *cbr1*∆ cells exposed to trehalose, maltose, and sucrose was indistinguishable or slightly (≤ 25%) higher than in wild-type cells, as expected given that *cbr1*∆ cells have no growth phenotype on these carbon sources (Figs 1C and 7C). However, when we added 244 µM 2-deoxyglucose to trehalose and maltose, *AGL1* expression was reduced by over 2.1-fold in *cbr1*∆ relative to wild-type cells (*p*_adj_ < 0.05, Welch’s *t* test) (Fig 7C). We also saw a 2.6-fold reduction in *AGL1* expression in *cbr1*∆ relative to wild-type cells when we added 7.6 µM 2-deoxyglucose to sucrose (*p*_adj_ < 0.05, Welch’s *t* test) (Fig 7C). Similarly, the expression of the predicted proline dehydrogenase gene (RTO4_14602), which was activated specifically in response to proline (*p*_adj_ < 0.05, two-way ANOVA with a TukeyHSD post-hoc test), was 2.1-fold lower in *cbr1*∆ than wild-type cells when 122 µM 2-deoxyglucose was added to proline (*p*_adj_ < 0.05, Welch’s *t* test) (Figs 7D and S14C).

Canonically, regulators of carbon catabolite repression regulate a broad spectrum of nonpreferred carbon source utilization genes [1,3–10]. We hypothesized *CBR1* would play a similar role in *R. toruloides*. However, the growth of *cbr1*Δ cells exposed to 2-deoxyglucose in combination with the pentose monosaccharide xylose was indistinguishable from wild-type cells (Figs 7E and S13C). To test whether carbon catabolite repression of xylose utilization was regulated by Cbr1 at the transcriptional level, we measured expression of the pentose reductase gene RTO4_9774 [54,55]. Corroborating the similarity in growth between wild-type and *cbr1*∆ cells on xylose with and without 2-deoxyglucose, we saw no difference in the expression of RTO4_9774 between wild-type and *cbr1*∆ cells on xylose alone or with any concentration of 2-deoxyglucose (Fig 7F). Similarly, the growth of wild-type and *cbr1*∆ cells was indistinguishable on the lipid mimic Tween-80 in combination with 2-deoxyglucose (Figs 7G and S13D). To measure the role of *CBR1* in regulating genes involved in alcohol utilization, we used allyl alcohol, which is converted to the toxic molecule acrolein by alcohol dehydrogenases. Alcohol dehydrogenase expression is repressed by glucose [56]. Wild-type and *cbr1*Δ cells were equally sensitive to allyl alcohol, indicating glucose-mediated repression of alcohol dehydrogenases is not dependent on Cbr1 (Figs 7H and S13E). To determine whether any *CBR1* core regulon genes played a role in carbon catabolite repression, we tested 2-deoxyglucose sensitivity of *bgl1*Δ *bgl2*Δ, *tct1*Δ, RTO4_10339Δ, and RTO4_11229Δ cells. The sensitivity of these strains to 2-deoxyglucose during exposure to trehalose, maltose, xylose, and tween-80 was indistinguishable from wild-type cells (S15 Fig). These data suggest Cbr1 inhibits carbon catabolite repression of disaccharides and proline either by directly regulating gene expression or by indirectly regulating disaccharide and proline utilization genes through genes outside of the *CBR1* core regulon.

A role for Cbr1 Ascomycete homologs in carbon catabolite repression regulation has not been identified, so we asked whether the *N. crassa* Cbr1 homolog CLR-2 plays a similar role. The sensitivity of wild-type and Δ*clr-2* cells to 2-deoxyglucose was indistinguishable during growth on the glucose-glucose disaccharide trehalose or the monosaccharide xylose (S16 Fig). These data indicate the role for Cbr1 in *R. toruloides* in directly or indirectly inhibiting carbon catabolite repression of disaccharides and proline is an expanded role for Cbr1 relative to the model Ascomycete ortholog CLR-2.

## Discussion

**Fig 8:**
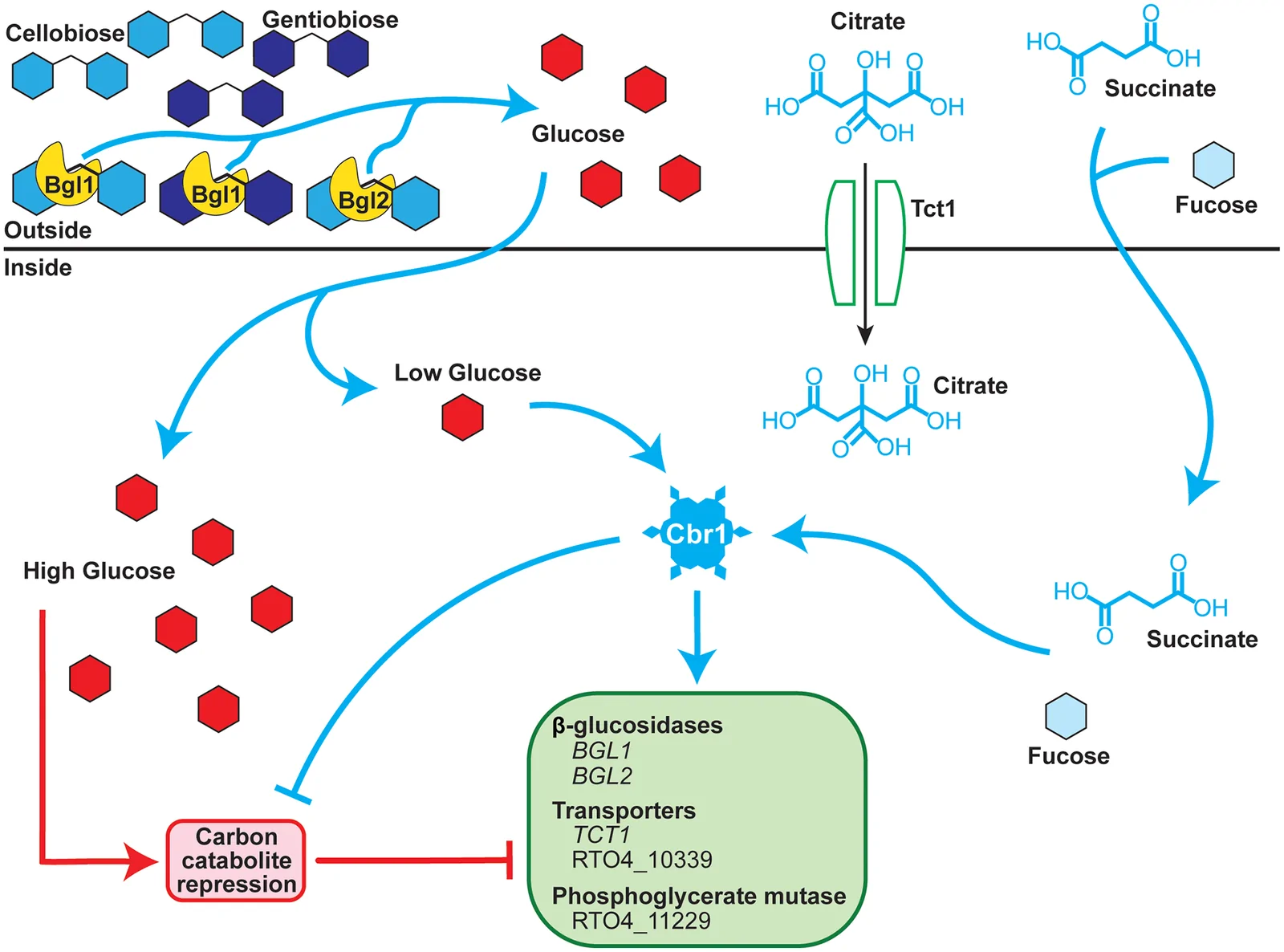
Model for Cbr1-mediated regulation of cellobiose, gentiobiose, TCA cycle intermediate, and fucose utilization and carbon catabolite repression. Cbr1 is activated by the presence of a low glucose concentration, fucose, and TCA cycle intermediates or their metabolic derivatives. Activated Cbr1 directly or indirectly induces the expression of genes encoding β-glucosidases, predicted transporters, and a predicted phosphoglycerate mutase. The Cbr1-regulated transporter gene *TCT1* is required for citrate import. Transporters for other TCA cycle intermediates and fucose are unknown. The β-glucosidase genes *BGL1* and *BGL2* encode secreted β-glucosidases that cleave cellobiose and gentiobiose into two glucose molecules. *BGL1* is required for growth on cellobiose. *BGL1* and/or *BGL2* are required for growth on gentiobiose. A high glucose concentration activates carbon catabolite repression, which represses β-glucosidase gene expression and likely represses the expression of genes necessary to utilize fucose and TCA cycle intermediates. Cbr1 plays a role in directly or indirectly inhibiting carbon catabolite repression, specifically when a disaccharide or proline is present as the nonpreferred carbon source.

Gene regulation in response to nutrient availability requires cells to integrate information from activating and repressing nutrient signals. Previously described fungal transcriptional networks regulating carbon utilization are made up of carbon source-specific transcription factors that activate genes necessary to utilize a particular carbon source when it is present and broad-acting transcription factors that regulate carbon catabolite repression [1,2]. However, studies of these carbon source utilization transcriptional networks have mainly focused on Ascomycete fungi. We investigated nutrient sensing regulatory mechanisms in a Basidiomycete yeast and identified a transcription factor that directly or indirectly coregulates carbon source-specific gene activation and carbon catabolite repression specifically of disaccharide and proline utilization genes (Fig 8).

### Cbr1 direct or indirect regulation of cellobiose, gentiobiose, TCA cycle intermediate, and fucose utilization may shed light on complex microbial interactions

There have been limited investigations of genetic regulators of cellulose and cellobiose utilization in Basidiomycetes. A recent study identified Roc1 as a regulator of cellulase-encoding genes in the basidiomycete wood-decaying fungus *Schizophyllum commune* [57]. However, homologs of Roc1 do not exist outside of the Agaricomycetes [57]. In *Ganoderma lucidum*, the R2R3-type MYB transcription factor *Gl*Myb activates cellulase gene expression [58]. In Ascomycete filamentous fungi, CLR-2/ClrB regulates cellulose degradation and utilization through direct activation of cellulase genes [37,59]. We identified the CLR-2/ClrB ortholog Cbr1 in the oleaginous, basidiomycete yeast *R. toruloides* (Fig 1). Although Ascomycete and Basidiomycete fungi are separated by approximately 400 million years of evolution [12], Cbr1 is required to utilize the cellulose breakdown product cellobiose (Fig 1). *N. crassa* CLR-2 does not directly regulate the expression of genes encoding β-glucosidases, which are regulated by CLR-1 [37,59]. However, although the role of Cbr1 is significantly expanded beyond that of CLR-2/ClrB to include inhibition of carbon catabolite repression and utilization of gentiobiose, TCA cycle intermediates, and fucose, Cbr1 direct or indirect regulation of cellobiose utilization indicates some functional conservation of CLR-2/ClrB/Cbr1 exists between Ascomycete filamentous fungi and Basidiomycete yeast.

Cbr1 directly or indirectly regulates citrate utilization through the transporter gene *TCT1* and cellobiose and gentiobiose utilization through the β-glucosidase genes *BGL1* and *BGL2* (Fig 8). Genetic mechanisms through which Cbr1 regulates utilization of fucose and dicarboxylic acid TCA cycle intermediates remains unclear. Carbon utilization genes tend to be highly upregulated in conditions in which they are necessary in fungi, and activation significantly higher than 4-fold is frequently observed [3,37,39,57]. Thus, we chose a 4-fold threshold to identify genes that were regulated by Cbr1 under a variety of conditions. However, it is possible that this 4-fold cutoff caused us to miss genes required for fucose or dicarboxylic acid utilization. It is also possible that the two uncharacterized genes in the *CBR1* core regulon, the predicted transporter RTO4_10339 and the predicted phosphoglycerate mutase RTO4_11229, may play roles in fucose and/or dicarboxylic acid utilization that are redundant with other *CBR1*-regulated genes that did not meet our strict *CBR1* core regulon criteria (Figs 2, S3, and S4). It will be the role of future studies to characterize roles for RTO4_10339, RTO4_11229, and other genes that may be responsible for fucose and dicarboxylic acid catabolism in the *CBR1* pathway.

In other fungal species, utilization of multiple carbon sources is occasionally regulated by a single transcription factor. CLR-2 in *N. crassa* regulates cellulose utilization, mannan utilization, and a small number of hemicellulose utilization genes [3,37,41,59]. In *Trichoderma reesei*, *Penicillium oxalicum*, and some Aspergilli, the transcription factor Xyr1/XlnR regulates cellulose and hemicellulose utilization [38–40]. ARA-1/Ara1 regulates arabinan and galactose utilization in *N. crassa* and *T. reesei* [3,60]. FarA regulates utilization of fatty acids and the lignin component ferulic acid in Aspergilli [61,62]. In *Candida albicans*, Adr1 is required for growth on citrate and substrates that feed into the TCA cycle, like glutamate and malate [63]. However, in these examples, linkage of these carbon sources can be explained either by the presence of both carbon sources in the plant cell wall (i.e., cellulose and hemicellulose or arabinan and galactose) or shared catabolic pathways (i.e., fatty acids and ferulic acid or citrate, glutamate, and malate).

Cellobiose/gentiobiose, fucose, and TCA cycle intermediates are not linked by known catabolic pathways, and our data do not support catabolism through the same noncanonical metabolic pathway in *R. toruloides*. Cbr1-regulated β-glucosidase genes are critical for cellobiose and gentiobiose but not fucose or TCA cycle intermediate utilization (Figs 3 and S6A), while Tct1 is necessary for citrate but not cellobiose or fucose utilization (Figs 6A and S10A). Aside from *CBR1*, we did not identify genes necessary for utilization of all four of cellobiose, gentiobiose, fucose, and TCA cycle intermediates.

We speculate coregulation of cellobiose, gentiobiose, fucose, and TCA cycle intermediate utilization may suggest *R. toruloides* encounters these carbon sources together in nature. Although many possible explanations exist, we posit it is possible *R. toruloides* scavenges cellobiose, gentiobiose, and fucose during plant biomass degradation by filamentous fungi, which secrete organic acids like citrate or succinate to acidify their environment and can contain fucose in their cell walls [64–66]. β-glucosidase gene activation that is primed by organic acids secreted by fungi or fucose in fungal cell walls before encountering cellobiose released from cellulose could confer a growth advantage to *R. toruloides* over microbes that only activate β-glucosidase genes in response to cellobiose itself. Indeed, cells constitutively expressing *BGL1* that do not require time to activate β-glucosidase genes had a growth advantage over wild-type cells that activated β-glucosidase genes in response to cellobiose or gentiobiose (Fig 4B and 4C). Potential mechanisms of adaptive prediction may not be limited to Cbr1-mediated regulation. We also observed trehalase activity in succinate supernatants in a manner that is only partially dependent on Cbr1 (S6F Fig), suggesting that other regulatory mechanisms in *R. toruloides* also exist to connect metabolically unrelated carbon utilization.

### Cbr1 direct or indirect regulation of carbon catabolite repression of disaccharides disrupts a negative feedback loop for disaccharide utilization

Carbon catabolite repression enables prioritization of carbon sources that require less energy to metabolize over carbon sources that require more energy to metabolize [1]. Known carbon catabolite repression genetic mechanisms regulate genes required for utilization of all less preferred carbon sources when a more preferred carbon source is present [3–10]. In contrast, Cbr1 directly or indirectly inhibits carbon catabolite repression specifically of disaccharide and proline utilization (Fig 7). We speculate this mechanism allows continued expression of genes that hydrolyze disaccharide bonds even when this hydrolysis produces preferred monosaccharides, combating a negative feedback loop (Fig 8). To our knowledge, genetic linkage via a single transcription factor of carbon catabolite repression inhibition with activation of genes necessary for nonpreferred carbon source utilization has not been described in fungi. Additionally, regulation of genes involved in utilization of specific classes of nonpreferred carbon sources in response to a preferred carbon source represents a previously uncharacterized form of carbon catabolite repression regulation.

Our data show that in response to glucose, Cbr1 directly or indirectly activates genes necessary for cellobiose, gentiobiose, and citrate utilization and inhibits repression of genes necessary for disaccharide and proline utilization, like *AGL1*, RTO4_14602, and, presumably, *BGL1*, *BGL2*, and trehalase genes. The direct or indirect targets of Cbr1 do not appear to include genes necessary for monosaccharide, lipid, or alcohol utilization in response to preferred carbon sources. However, the molecular mechanism through which this regulation is achieved is unclear. Since Cbr1 is neither regulated through expression levels nor nuclear localization, this suggests that Cbr1 activity may be regulated through conformational changes enabling activation of target genes.

In Ascomycete fungi, the transcriptional repressor Mig1/CRE-1/CreA localizes to the nucleus when preferred carbon sources are available where it binds the promoters of genes necessary to utilize nonpreferred nutrient sources and represses their expression [3,5,6,67–71]. In the absence of preferred carbon sources, the AMP-activated kinase Snf1 is thought to phosphorylate Mig1/CRE-1/CreA, sequestering it in the cytoplasm and relieving transcriptional repression [1,72–76]. The molecular mechanisms mediating carbon catabolite repression in Basidiomycetes are not well studied. Only the DNA-binding domain is conserved in the closest homolog to Mig1 or CRE-1/CreA in *R. toruloides* (RTO4_10798), and future studies will be necessary to identify the role of RTO4_10798 or other transcriptional repressors in regulating carbon catabolite repression in *R. toruloides* and other Basidiomycetes.

We predict substrate-specific regulation of carbon catabolite repression in *R. toruloides* is achieved in one of two ways. One possibility is that a network of transcription factors, which includes Cbr1, each have promoter binding sites in genes necessary to utilize different classes of nonpreferred carbon sources that modulate gene expression through direct interactions with RNA polymerase, changing gene expression levels, or blocking binding by other activating or repressing transcription factors. Alternatively, Cbr1 could act indirectly on disaccharide and proline utilization genes by repressing expression of carbon catabolite repressing transcription factors when disaccharides or proline are present. However, other possible mechanisms of substrate-specific carbon catabolite repression exist, including compartmentalization at the level of transporters, receptors, or signaling cascades. It will be the role of future studies to place Cbr1 in a network of receptors, signaling cascades, and transcription factors regulating preferred and nonpreferred carbon source utilization genes.

Fungal pathogens of plants and animals that do not regulate carbon catabolite repression like wild-type cells frequently have reduced virulence [8,77–79]. Carbon utilization and carbon catabolite repression regulation by fungi also affects how they respond to antifungal drugs [14,15]. Thus, understanding diverse carbon catabolite repression mechanisms present throughout the fungal kingdom is critical in controlling fungal disease. Additionally, a more complete understanding of mechanisms of carbon catabolite repression and the diversity of nutrient sensing transcriptional networks is necessary for harnessing the primary and secondary metabolic capabilities of the fungal kingdom for biotechnology. Mapping these networks will be critical in metabolically engineering fungi for green industrial fermentation.

## Materials and methods

### *R. toruloides* strains and culturing

*R. toruloides* strains used in this study are listed in S1 Table. All strains were derived from the wild-type reference strain IFO 0880 (also called NBRC 0880, obtained from Biological Resource Center, NITE (NBRC), Japan). The starting strain for genetic manipulations was the non-homologous end-joining deficient *yku70*Δ (RTO4_11920∆) strain [80]. *R. toruloides* was transformed using *Agrobacterium tumefaciens*-mediated transformation as previously described [31], using *A. tumefaciens* EHA 105 and plasmids derived from pGI2 [81]. Most gene deletions, complementations, constitutive expressions, and tags were performed using homologous recombination of a nourseothricin or G418 resistance cassette with flanking arms of ~1,000 bp. Nourseothricin (GoldBio) and G418 (VWR) were both added to selection media at 100 µg/mL.

There were a few specialized cases of strain construction. We fluorescently tagged the histone H2A subunit gene *HTA1* (RTO4_12090) with the mRuby2 fluorescent protein at the native locus. The Hta1-mRuby2 tagged strains were generated using a combination of G418 resistance and a 5-fluorodeoxyuridine (FUDR) sensitive thymidine kinase counterselection marker flanked by 250 bp terminal repeat sequences. The Hta1-mRuby2 construct was transformed into the *CBR1*-*GFP* background using G418 to select for transformants. Transformed colonies were passaged one time on liquid yeast peptone dextrose (YPD) (VWR) and plated onto yeast nitrogen base (YNB) with (NH_4_)_2_SO_4_ without amino acids (MP Biomedicals) + 1% glucose + 50 µg/mL FUDR (VWR) plates to select for loop-outs of the G418 and thymidine kinase markers. Colonies were screened by microscopy, PCR, and locus sequencing for correct integration of the mRuby2 tag and successful removal of the G418 and thymidine kinase markers. Since RTO4_16716 and RTO4_16717 are adjacent in the genome, the *bgl1*Δ *bgl2*∆ strain was generated via single transformation of the nourseothricin resistance cassette to remove both open reading frames from the genome, and the *bgl1∆ bgl2∆ + BGL1 BGL2* complement strain was generated from the *bgl1∆ bgl2∆* strain by transforming a single genomic fragment containing both coding sequences. The *cbr1∆ + P*_*AAC1*_*-BGL1* strain was generated via transformation of the *BGL1* open reading frame driven by the previously characterized constitutive *AAC1* (RTO4_12704) promoter [47] and the 35S rRNA terminator into the *YKU70* locus with a G418 resistance cassette for selection. To generate the *tct1*∆ + *P*_*AAC1*_*-mRuby2-TCT1* strains, we fluorescently tagged the Tct1 N-terminus with mRuby2 and constitutively expressed the *mRuby2-TCT1* construct using the *AAC1* promoter and the *nos* terminator. The *P*_*AAC1*_*-mRuby2-TCT1* construct, with a nourseothricin resistance cassette for selection, was transformed into the *YKU70* locus of the *tct1*∆ strain. All strains were confirmed by PCR. Complement, tagged, and constitutive expression strains were further confirmed by DNA sequencing of the transformed loci.

*R. toruloides* strains were grown from freezer stocks on YPD (VWR) + 2% agar (BD Difco Bacto) plates at 28 °C for 2–3 days prior to inoculation into liquid YPD to grow strains at 28 °C with constant shaking at 250 rpm to exponential phase growth prior to starting experiments. All *R. toruloides* experiments were performed with either YNB with (NH_4_)_2_SO_4_ without amino acids (MP Biomedicals) + 12 mM K_2_HPO_4_ (Fisher) + 38 mM KH_2_PO_4_ (Fisher) + 100 nM Fe(II)SO_4_ pH 6.2 or YNB without (NH_4_)_2_SO_4_ or amino acids (BD Difco) + 38 mM (NH_4_)_2_SO_4_ (VWR) + 12 mM K_2_HPO_4_ (Fisher) + 38 mM KH_2_PO_4_ (Fisher) + 100 nM Fe(II)SO_4_ pH 6.2 with the indicated carbon source at 1% w/v with the exception of coumaric acid and ferulic acid, which were added at 0.2% w/v, citrate which was added at 8.5 mM, and gentiobiose which was added at 10 mM. Carbon sources used are listed in S3 Table.

### *N. crassa* strains and culturing

*N. crassa* strains used in this study are listed in S2 Table. These strains were derived from the wild-type reference strain FGSC 2489 and obtained from the Fungal Genetics Stock Center [82,83]. *N. crassa* cells were grown from freezer stocks on Vogel’s minimal medium [84] + 2% sucrose + 1.5% agar (BD Difco Bacto) slants for 2 d at 28 °C in the dark and 4 d at 28 °C in constant light. *N. crassa* conidia were harvested from slants with sterile double-distilled H_2_O for inoculation. All *N. crassa* experiments were performed using Vogel’s minimal medium [84] with 50 mM NH_4_Cl instead of 25 mM NH_4_NO_3_ (VMM NH_4_Cl) and the indicated carbon source at 1% w/v. Carbon sources used are listed in S3 Table.

### *R. toruloides* growth experiments

*R. toruloides* colonies grown on solid YPD were inoculated into liquid YPD and grown at 28 °C with constant shaking at 250 rpm to exponential phase. Cells were then washed three times with YNB lacking a carbon source and inoculated into 750 μL of the indicated media in 48-well plates at the following starting densities, as measured by an Eppendorf BioPhotometer with a 10 mm path length: 0.1 OD600/mL for initial carbon screening experiments (Fig 1C–1F) of wild type and *cbr1*∆ in YNB 1% acetate, YNB 1% arabinose, YNB 1% fructose, YNB 1% galacturonic acid, YNB 1% glucose, YNB 1% glutamate, YNB 1% glycine, YNB 1% L-alanine, YNB 1% maltose, YNB 1% Tween-80, and YNB 1% xylose; 0.5 OD600/mL for the high density *cbr1*∆ + *P*_*AAC1*_*-BGL1* growth experiment (Fig 4B); and 0.01 OD600/mL for all other growth experiments. Forty-eight-well plates were incubated at 28 °C with constant shaking (3 mm orbit, 600 rpm for manual, individual time points, except for Fig 7G, which was shaken at 430 rpm; and 3 mm orbit, 425 rpm for kinetic growth experiments). Growth was measured as OD600 on an Agilent BioTek Synergy HTX for manual, individual time points or an Agilent BioTek Epoch2 for kinetic growth experiments with OD600 measurements taken every 20 or 30 min depending on the experiment.

### *N. crassa* growth experiments

For growth of *N. crassa* on trehalose and xylose with or without 2-deoxyglucose (TCI) wild-type *mat A* (FGSC 2489) and Δ*clr-2 mat A* conidia were inoculated at 10^6^ conidia/mL into 250 mL flasks with 100 mL of filter-sterilized VMM NH_4_Cl with either 1% xylose or 1% trehalose. Filter-sterilized 20% 2-deoxyglucose dissolved in water was added to the media to achieve 2-deoxyglucose concentrations of 0 μM, 119 μM, 238 μM, or 476 μM 2-deoxyglucose for media containing xylose as the sole carbon source and 2-deoxyglucose concentrations of 0 mM, 2.4 mM, 3.8 mM, or 7.6 mM for media containing trehalose as the sole carbon source. Cultures were grown at 28 °C with constant light and shaking at 200 rpm. After 24 h, biomass was autoclaved, harvested via filtration, washed with distilled water, dried for 24 h at 60 °C in a drying oven, and weighed.

For growth of *N. crassa* on tricarboxylic acid (TCA) cycle intermediates and fucose, wild-type *mat A* (FGSC 2489) and ∆*clr-2 mat A* conidia were inoculated at 10^6^ conidia/mL in 24-well deep-well round-bottomed plates with 3 mL of filter-sterilized VMM NH_4_Cl with 1% succinate, 1% α-ketoglutarate, 1% malate, or 1% fucose. Cultures were incubated at 28 °C with constant light and shaking (200 rpm). Biomass from TCA cycle intermediates and fucose cultures was harvested by filtration 7 and 10 days post-inoculation, respectively. Biomass was washed with distilled water, dried for 24 h in a drying oven at 60 °C, and weighed.

### Phylogenetic tree generation

The Basic Local Alignment Search Tool for proteins (BLASTP) was used to identify homologs of CLR-1, CLR-2, and XLR-1 from *N. crassa* (OR74A) and ClrA, ClrB, and XlnR from *Aspergillus nidulans* (A4) in the proteomes of *N. crassa*, *A. nidulans*, *S. cerevisiae* (S288C)*,* and *R. toruloides* (IFO 0880). CLR-1, CLR-2, and XLR-1 from *N. crassa* (OR74A) and ClrA, ClrB, and XlnR from *A. nidulans* (A4) protein sequences were obtained from Mycocosm [85]. Homologs with *E* < 10^−10^ were aligned via MUSCLE [86] in MEGA11 [87] and the phylogenetic tree was generated using FastTree [88] using the maximum likelihood method based on the Jones–Taylor–Thornton matrix-based model. The reliability of each split in the tree was computed using the Shimodaira–Hasegawa test on three alternate topologies around that split. Trees were visualized in MEGA11 [87] and rooted on the midpoint.

### Microscopy

Fluorescent microscopy was performed using a Nikon Eclipse Ti2-E inverted microscope equipped with a prime BSI express CMOS camera using a CFI Plan Apochromat 100× oil objective. Exponentially growing cells were pelleted at 4,000*g* and washed three times with YNB without a carbon source. For Cbr1-GFP localization, washed *HTA1-mRuby2 CBR1-GFP* cells were inoculated into a 48-well plate with YNB + 1% w/v of the indicated carbon source at 0.2 OD600/mL, as measured by an Eppendorf BioPhotometer with a 10 mm path length, and incubated at 28 °C with shaking (3 mm orbit, 600 rpm) for 4 h. Aliquots of the cultures were placed onto agarose-coated slides and imaged.

Localization of constitutively expressed Tct1-mRuby was determined by inoculating washed *P*_*ACC1*_*-mRuby-TCT1* cells into a 48-well plate with YNB with the indicated carbon source (at 1% for all carbon sources except citrate, which was at 8.5 mM) at 0.01 OD600/mL, as measured by an Eppendorf BioPhotometer with a 10 mm path length, and incubated at 28 °C with shaking (3 mm orbit, 600 rpm). Cultures were monitored for growth via OD600 measurements on an Agilent BioTek Synergy HTX plate reader. When cells reached exponential phase, a 200 µL aliquot of *P*_*ACC1*_*-mRuby-TCT1* cells was harvested for staining and imaging. Cells were stained with 25 µg/mL calcofluor white (fluorescent brightener 28, Sigma-Aldrich) 5 min before imaging. Citrate and fucose-grown cells were also stained with 400 nM Mitotracker Green FM (Invitrogen) 15 min prior to imaging. Aliquots of the stained cells were placed onto agarose-coated slides and imaged.

### RNA sequencing and transcript abundance

Cells were grown to exponential phase in liquid YPD and washed three times in YNB without a carbon source. Two hundred fifty mL flasks containing 100 mL of YNB without a carbon source or YNB with the indicated carbon source were inoculated with the indicated exponentially growing cells at 0.1 OD600/mL, as measured by an Eppendorf BioPhotometer with a 10 mm path length. Culture conditions and sequencing methods for each dataset are summarized in S4 Table. Cultures were incubated at 28 ºC with constant shaking (200 rpm) for the following times: 8 h for wild-type and *cbr1*∆ cells exposed to YNB 1% cellobiose, YNB 1% succinate, YNB 1% fucose, and YNB without a carbon source (Fig 2), 4 h for wild-type, *cbr1*∆, and *bgl1*∆ *bgl2*∆ cells exposed to YNB 1% cellobiose (Fig 5), 24 h for wild-type, *cbr1*∆, and *tct1*∆ cells exposed to YNB 8.5 mM citrate (Fig 6E), 24 h for wild-type cells exposed to YNB 1% succinate (used to construct GFF file with improved 3′ untranslated regions [UTR] [S6 Data]), and 8 h for wild-type cells exposed to VMM NH_4_Cl 2% glucose (used to construct GFF file with improved 3′ UTRs [S6 Data]).

Cells were harvested using centrifugation in 50 mL conical tubes at 4,000*g*. The supernatants were removed, and the cell pellets were flash frozen in liquid nitrogen. RNA was extracted using the Quick-RNA Fungal/Bacterial Miniprep Kit (Zymo) according to the manufacturer instructions, followed by either treatment with the TURBO DNA-free kit (Invitrogen) for standard RNAseq or DNase I (NEB) treatment for 10 min at 37 °C and purification with the Monarch Spin RNA Cleanup Kit (NEB) for 3′ RNAseq. RNA quality was assessed via agarose gel electrophoresis, and a subset of samples were also assessed for RNA quality using an Agilent BioAnalyzer at the Biotechnology Resource Center at Cornell University.

For wild-type and *cbr1*∆ cells exposed to YNB 1% cellobiose, YNB 1% succinate, and YNB without a carbon source for 8 h, RNA was submitted to the California Institute for Quantitative Biosciences at UC Berkeley (QB3-Berkeley) for library preparation and sequencing. Libraries were prepared using standard Illumina protocols and RNA was sequenced using an Illumina NextSeq 2000 with 150 bp paired end reads at a depth of approximately 20 million reads per sample. Raw reads were trimmed using fastp v. 0.23.4 with the --trim_poly_x setting [89]. The transcript abundance (transcripts per million, TPM) was quantified using Salmon v. 1.10.0 mapping to the *R. toruloides* IFO 0880 transcriptome (v4) [31] using the --validateMappings and --GCbias settings [90]. Differential expression was determined using DESeq2 v. 1.50.1 [91]. Genes were designated as differentially expressed if they had an average TPM greater than 1 in at least one of the two compared conditions, *p*_adj_ < 0.05, and log_2_(fold change) greater than 2 or less than −2. These RNAseq data were deposited in the Gene Expression Omnibus (GEO) at the National Center for Biotechnology Information (NCBI) and are accessible through GEO series accession number GSE293943.

For wild-type and *cbr1*∆ cells exposed to YNB 1% fucose; wild-type, *cbr1*∆, and *bgl1*∆ *bgl2*∆ cells exposed to YNB 1% cellobiose; wild-type, *cbr1*∆, and *tct1*∆ cells exposed to YNB 8.5 mM citrate; wild-type cells exposed to YNB 1% succinate for 24 h; and wild-type cells exposed to VMM NH_4_Cl 2% glucose for 8 h, RNA was submitted to the Biotechnology Resource Center at Cornell University for library preparation and sequencing. Libraries were prepared using the Lexogen QuantSeq 3′ mRNA-Seq Library Prep Kit FWD V2, and RNA was sequenced using an Illumina NovaSeqX with 150 bp paired-end reads at a depth of approximately 10 million reads per sample, except for wild type exposed to VMM NH_4_Cl 2% glucose for 8 h, which was sequenced on an Illumina NextSeq 500 with 75 bp single-end reads at a depth of approximately 7.5 million reads per sample. Raw reads were trimmed using fastp v. 0.23.4 with the --trim_poly_x setting [89]. Reads were aligned to the *R. toruloides* IFO 0880 genome (v4) [31] using STAR v. 2.7.11b [92] with --alignIntronMin set to 5, and BAM files were indexed using Samtools v. 1.20 [93].

Because the 3′ UTRs were not perfectly annotated in the *R. toruloides* IFO 0880 genome (v4) [31], leading to undercounting 3′ RNAseq reads, we used peaks2utr v. 1.4.1 [94] to annotate missing or truncated 3′ UTRs. To improve 3′ UTR annotation, we used all the wild type 3′ RNAseq conditions (YNB 1% fucose, YNB 1% cellobiose, YNB 8.5 mM citrate, YNB 1% succinate [24 h], and VMM NH_4_Cl 2% glucose). All the BAM files output by STAR v. 2.7.11b were combined, and the resulting combined BAM file was used as the peaks2utr input. In total, 2,696 3′ UTRs were annotated by peaks2utr and the resulting gff3 file is available in S6 Data. GNU parallel v. 20170522 [95] was used to run HTSeq v. 2.0.9 [96] in parallel for read counting using the peaks2utr generated gff3 (S6 Data) file with --nonunique set to random, -t set to mRNA, and -i set to ProteinId. Differential expression was determined using DESeq2 v. 1.50.1 [91]. Counts per million (CPM) values were determined by dividing the counts for a given gene by the sum of all counts for the sample and multiplying by 10^6^. Genes were designated as differentially expressed if they had an average CPM greater than 1 in at least one of the two compared conditions, *p*_adj_ < 0.05, and log_2_(fold change) greater than 2 or less than −2. Euclidean distance and hierarchical clustering were performed on the log_2_-transformed CPM for all genes that had a *p*_adj_ < 0.05 in at least one pairwise comparison and an average CPM of at least 1 in one of the three strains for wild type, *cbr1*∆, and *bgl1*∆ *bgl2*∆ exposed to cellobiose (S9A Fig; S7 and S8 Data). Expression data for wild-type, *cbr1*∆, and *tct1*∆ cells exposed to YNB 8.5 mM citrate was visualized using the ComplexHeatmap R package (Fig 6E) [97]. These RNAseq data were deposited in the GEO at NCBI and are accessible through GEO series accession number GSE313844.

All RNAseq experiments were performed with three biological replicates

### Functional enrichment analysis

Kyoto Encyclopedia of Genes and Genomes (KEGG) [43] and Gene Ontology (GO) [44,45] enrichment analyses were performed on genes differentially expressed between *cbr1*∆ and wild-type cells exposed to cellobiose, succinate, carbon starvation, and fucose (Fig 2) using the enricher function of the clusterProfiler R package v. 4.18.1 with Benjamini-Hochberg multiple hypothesis testing correction and visualized using the clusterProfiler dotplot function [98]. GO annotations and protein sequences for the *R. toruloides* IFO 0880 genome (v4) [31] were downloaded from Mycocosm (https://mycocosm.jgi.doe.gov/Rhoto_IFO0880_4/Rhoto_IFO0880_4.home.html) [85]. KEGG Orthology IDs were assigned to protein sequences using BlastKOALA [99] and mapped to KEGG pathways [43] using the clusterProfiler gson_KEGG_mapper function [98]. Pathways that are not biologically relevant to fungi were manually removed (e.g., Insect hormone biosynthesis, Olfactory transduction, etc.). Genes were included in the functional enrichment analysis input if they were at least 4-fold decreased in expression in the *cbr1*Δ strain relative to the wild-type strain with *p*_adj_ < 0.05 and either an average TPM or CPM of at least 1 in at least one condition. BlastKOALA [99] assignments are available in S11 Data.

### Reverse transcription quantitative PCR

To measure *BGL1* expression in wild-type cells during exposure to YNB 1% cellobiose, YNB 1% succinate, YNB 1% glucose, and YNB without a carbon source (Fig 3D), wild-type cells were grown in YNB 1% glucose overnight, back-diluted into fresh YNB 1% glucose, and grown for 6–7 generations until reaching an OD600 ~1. Cells were then washed twice in YNB without a carbon source and inoculated at 0.8 OD600/mL, as measured by an Agilent BioTek Synergy HTX plate reader, in 100 mL YNB 1% cellobiose, YNB 1% succinate, YNB 1% glucose, or YNB without a carbon source in 250 mL flasks. Cultures were incubated at 28 °C with shaking at 200 rpm for 4 h. 300 µL of culture was then harvested, pelleted at 5,500*g*, supernatants were removed, and cells were flash frozen in liquid N_2_ and stored at −80 °C until RNA was extracted.

To measure *BGL1* expression in wild-type cells during exposure to YNB lacking a carbon source, YNB 1% cellobiose, YNB 1% 2-deoxyglucose, YNB 100 µM 2-deoxyglucose, YNB 10 µM 2-deoxyglucose, YNB 1 µM 2-deoxyglucose, and YNB 0.1 µM 2-deoxyglucose (Fig 5D), exponentially growing cells were washed three times in YNB without a carbon source and 0.222 OD600/mL wild-type cells, as measured by an Eppendorf BioPhotometer with a 10 mm path length, were inoculated in 90 mL of YNB without a carbon source. Cultures were incubated at 28 °C with shaking at 200 rpm for 1 h. After 1 h, we added 10 mL of either YNB without a carbon source or YNB with 10× the final indicated concentrations of cellobiose or 2-deoxyglucose. Cultures were then incubated at 28 °C with shaking at 200 rpm. After 1 h, 10 mL of each culture was harvested, and after 4 h, 50 mL of each culture was harvested. Harvested cells were pelleted at 4,000*g*, the supernatants were removed, and cells were flash frozen in liquid N_2_ and stored at −80 °C until RNA was extracted.

To measure expression of *AGL1* and RTO4_14602 across a variety of carbon sources, we washed exponentially growing wild-type and *cbr1*∆ cells in YNB without a carbon source and inoculated them at 0.1 OD600/mL, as measured by an Eppendorf BioPhotometer with a 10 mm path length, into 100 mL of YNB lacking a carbon source, YNB 1% maltose, YNB 1% trehalose, YNB 1% sucrose, YNB 1% xylose, YNB 1% proline, and YNB 1% glucose. Cultures were incubated at 28 °C with shaking at 200 rpm for 8 h (for all carbon sources) and 24 h (sucrose only). Cells were then pelleted at 4,000*g*, supernatants were removed, and cells were flash frozen in liquid N_2_ and stored at −80 °C until RNA was extracted.

To quantify the impact of 2-deoxyglucose on expression of *AGL1*, RTO4_9774, and RTO4_14602, we washed exponentially growing wild-type and *cbr1*∆ cells in YNB without a carbon source and inoculated them at 0.1 OD600/mL, as measured by an Eppendorf BioPhotometer with a 10 mm path length, into 100 mL of YNB + 1% of the indicated carbon source (maltose, trehalose, and sucrose for *AGL1*; xylose for RTO4_9774; and proline for RTO4_14602) with the indicated concentration of 2-deoxyglucose. Cultures were incubated at 28 °C with shaking at 200 rpm for 8 h (for YNB 1% maltose, YNB 1% trehalose, YNB 1% xylose, and YNB 1% proline) or 24 h (for YNB 1% sucrose). Following the incubation, cultures were pelleted at 4,000*g*, supernatants were removed, and cells were flash frozen in liquid N_2_ and stored at −80 °C until RNA was extracted.

RNA was extracted using the Quick-RNA Fungal/Bacterial Miniprep Kit (Zymo) according to manufacturer instructions. Purified RNA was treated with DNase I (NEB) for 10 min at 37 °C. RNA was then purified using the Monarch Spin RNA Cleanup Kit (NEB). RNA quality was assessed via agarose gel electrophoresis. RT-qPCR was performed using the Luna Universal One-Step RT-qPCR Kit (NEB) according to manufacturer instructions with either 25 ng (carbon source experiment; Fig 3D) or 50 ng (2-deoxyglucose experiments; Figs 5D, 7C, 7D, 7F, S14A, and S14C) RNA as template. Data were collected on a CFX96 Real-Time System (Bio-Rad). Gene expression was normalized to *ACT1* (RTO4_14107) expression for all experiments. RT-qPCR primers are in S5 Table.

### β-glucosidase enzyme activity assays

Cells were grown to exponential phase in liquid YPD and washed three times in YNB without a carbon source. Washed cells were inoculated into 750 µL YNB 1% glucose, YNB 1% cellobiose, or YNB 1% succinate in 48-well plates at 0.8 OD600/mL, as measured by an Eppendorf BioPhotometer with a 10 mm path length. Wild-type, *cbr1*∆, *cbr1*∆ + *CBR1*, *bgl1*∆ *bgl2*∆, *bgl1*∆ *bgl2*∆ + *BGL1 BGL2*, *bgl1*∆, *bgl1*∆ + *BGL1*, and *bgl2*∆ cells were incubated for 20 h at 28^o^C with shaking (600 rpm, 3 mm orbit) (Figs 3C, S6B, and S6C). To measure the secreted β-glucosidase activity of *cbr1*∆ + *P*_*AAC1*_*-BGL1* cells (S7B Fig), wild-type and *cbr1*∆ + *P*_*AAC1*_*-BGL1* cells were incubated at 28 °C with shaking (600 rpm, 3 mm orbit) for 16 h to ensure both wild-type and *cbr1*∆ + *P*_*AAC1*_*-BGL1* cultures were in exponential phase at the time of the supernatant harvest. After 20 h or 16 h incubation, as described above, cultures were transferred to 1.5 mL Eppendorf tubes, and cells were pelleted by centrifuging at 14,000*g* for 3 min. Supernatants were transferred to 0.2 µm centrifuge tube filters (CoStar Spin-X) and centrifuged at 14,000*g* for 1 min. Filtered supernatants were stored at −20 °C.

β-glucosidase activity was determined using a p-nitrophenyl-β-D-glucopyranoside (EMD Millipore Corp.) colorimetric enzyme activity assay. We added 50 μL of 100 mM sodium acetate (pH 5) and 25 μL of freshly prepared 20 mM p-nitrophenyl-β-D-glucopyranoside to each well of a 96-well plate on ice. We then added 25 µL of each supernatant to the wells of the 96-well plate and mixed by pipetting (Figs 3C, S6E, S7B, and S7C). To better determine whether β-glucosidase activity was present in *bgl1*∆ supernatants, we mixed 25 µL of 100 mM sodium acetate (pH 5) and 25 µL of freshly prepared 20 mM p-nitrophenyl-β-D-glucopyranoside dissolved in 100 mM sodium acetate (pH 5) and then added 50 µL of the indicated supernatant (S6C Fig). In each assay, an equal number of mock reaction wells lacking supernatant were also prepared. Reactions were incubated at 37 °C for 35 min followed by quenching with 100 µL of 200 mM Na_2_CO_3_. After quenching, 25 µL (Figs 3C, S6E, S7B, and S7C) or 50 µL (S6C Fig) of supernatant were added to the mock reactions as blanks for their respective reactions. Absorbance was measured at 400 nm (OD400) using an Agilent BioTek Synergy HTX plate reader. To calculate p-nitrophenyl released from p-nitrophenyl-β-D-glucopyranoside, we generated a standard curve of OD400 values of 2-fold dilutions of p-nitrophenyl in the same buffer we used for the β-glucosidase activity assay (i.e., 200 µl of 25 mM sodium acetate (pH 5), 100 mM Na_2_CO_3_, and 0.125 × YNB without a carbon source).

### Glucose release assays

Exponentially growing wild-type, *cbr1*∆, *bgl1*∆, *bgl2*∆, and *bgl1*∆ *bgl2*∆ cells were washed three times with YNB without a carbon source and inoculated at 0.1 OD600/mL, as measured by an Eppendorf BioPhotometer with a 10 mm path length, in 100 mL of YNB 1% succinate in 250 mL flasks. Cultures were shaken at 200 rpm at 28 °C until they reached an OD600 of ~0.7 as measured by an Agilent BioTek Synergy HTX plate reader. We then centrifuged the cultures at 4,000*g* for 5 min, sterilized the supernatants with vacuum bottle-top 0.22 µm filters (VWR), and stored the sterilized supernatants at 4 °C for up to three days until all cultures reached the desired OD600. To measure activity of the culture supernatants, we mixed 15 µL of sterilized supernatant with 15 µL of filter-sterilized YNB 2% cellobiose, YNB 2% gentiobiose, YNB 2% maltose, YNB 2% trehalose, YNB 2% sucrose, or YNB 2% lactose and incubated for 48 h at 28 °C. As blanks for each carbon source, we also mixed 15 µL of YNB without a carbon source with 15 µL of filter-sterilized YNB 2% cellobiose, YNB 2% gentiobiose, YNB 2% maltose, YNB 2% trehalose, YNB 2% sucrose, or YNB 2% lactose, respectively, and incubated for 48 h at 28 °C. After the 48 h incubation, supernatant/YNB sugar mixtures were heat-shocked in a 70 °C water bath for 1 h. We assessed glucose released from each disaccharide using the Megazyme D-glucose hexokinase assay (Megazyme, K-GLUHK-220A) in a clear flat-bottomed 96-well plate according to the manufacturer’s instructions. To confirm β-glucosidase activity in culture supernatants, we also assessed p-nitrophenyl release from p-nitrophenyl-β-D-glucopyranoside as described above.

### Spent media growth assays

Exponentially growing wild-type cells were washed three times with YNB without a carbon source and inoculated at 0.01 OD600/mL, as measured by an Eppendorf BioPhotometer with a 10 mm path length, in 100 mL YNB 1% glucose, YNB 1% cellobiose, or YNB 1% succinate in 250 mL flasks. Cultures were grown to an OD600 of ~1, as measured by an Agilent BioTek Synergy HTX plate reader, prior to centrifugation at 4,000*g* for 10 min. The supernatants were sterilized with vacuum bottle-top 0.22 µm filters (VWR). The β-glucosidase activity of each supernatant was assessed using the β-glucosidase assay described above. Filter-sterilized supernatants were added to YNB lacking a carbon source or YNB 1% cellobiose to a final concentration of 6%, 12%, or 25% spent supernatant. Exponentially growing wild-type, *cbr1*∆, and *bgl1*∆ *bgl2*∆ cells were washed three times with YNB without a carbon source and inoculated at 0.01 OD600/mL, as measured by an Eppendorf BioPhotometer with a 10 mm path length, in 750 µL of media containing the indicated concentrations of filtered supernatant in 48-well plates. Cultures were incubated at 28 °C with shaking at 600 rpm (3 mm orbit), and OD600 measurements were taken at the indicated times. To control for growth on any remaining carbon or waste products in the spent supernatant, growth was normalized by subtracting the OD600 value of a paired replicate grown in spent supernatant-supplemented YNB no carbon from spent supernatant-supplemented YNB 1% cellobiose.

### Citrate uptake assay

Exponentially growing cells were washed three times with a pH 6.2 citrate-phosphate buffer (8.5 mM trisodium citrate [VWR] + 12 mM K_2_HPO_4_ [Fisher] + 38 mM KH_2_PO_4_ [Fisher]) and inoculated into 250 mL flasks containing 100 mL of either YNB + 8.5 mM citrate or citrate-phosphate buffer at an OD600/mL of 2. Cultures were incubated at 28 °C with shaking at 200 rpm. One mL aliquots were harvested 0, 1, 4, 6, and 8 days after inoculation for YNB + 8.5 mM citrate cultures and 7, 8, 9, 10, and 11 days after inoculation for citrate-phosphate buffer cultures. Cultures were centrifuged at 13,000*g* for three minutes to pellet cells, and the top 700 µL of supernatant was transferred to an Eppendorf tube and stored at −20 °C until citrate was quantified. Supernatants were thawed and diluted 1:10 in ddH_2_O. Citrate was quantified using a colorimetric citrate assay kit (Abcam) according to the manufacturer’s instructions.

### Statistical significance tests

All statistical analyses were performed in Rstudio (v2025.09.2 + 418; R v4.5.2) using either a one-way or two-way ANOVA with a Tukey post-hoc test (bar graphs), a two-tailed Welch’s two-sample *t* test (bar graphs), or a general linear model with two-tailed post-hoc analysis using the emmeans function of the emmeans R package [100] with a Holm multiple comparison correction (line graphs). Statistical significance for differential expression of RNAseq data was determined using DESeq2 [91]. All RNAseq experiments were performed with three biological replicates. All other experiments were performed with a minimum of three biological replicates with the exact number of replicates indicated in the relevant figure legend and in S3 Data. Exact adjusted *p*-values for each comparison are in S3 Data.

## Supporting information

S1 Fig Phylogenetic tree of homologs of transcription factors regulating cellulose and hemicellulose utilization. Phylogenetic tree of CLR-1/ClrA, CLR-2/ClrB, and XLR-1/XlnR homologs in *Rhodotorula (Rhodosporidium) toruloides*, *Neurospora crassa*, *Aspergillus nidulans*, and *Saccharomyces cerevisiae* built using the maximum likelihood method based on the Jones–Taylor–Thornton matrix-based model using FastTree [88]. Numbers at nodes indicate the reliability of each split in the tree computed using the Shimodaira–Hasegawa test on three alternate topologies around that split with 1,000 resamples. *R. toruloides* proteins are indicated with maroon diamonds. *N. crassa* proteins are indicated with orange squares. *A. nidulans* proteins are indicated with green circles. *S. cerevisiae* proteins are indicated with blue triangles. Scale bar indicates amino acid substitutions per site. The tree file for this phylogenetic tree can be found in S12 Data.
(TIF)

S2 Fig *N. crassa clr-2* is not required for utilization of TCA cycle intermediates or fucose. Mycelial dry weight of wild-type (WT) and *∆clr-2 N. crassa* cells directly inoculated into 3 mL of (**A**) VMM NH_4_Cl 1% TCA cycle intermediates grown for 7 days post-inoculation or (**B**) VMM NH_4_Cl 1% fucose grown for 10 days post-inoculation. Bars are the average and dots are the individual data points of three biological replicates. The data underlying this figure can be found in S2 Data.
(TIF)

S3 Fig Exposure to cellobiose and succinate yield a similar transcriptional profile that is largely independent of *CBR1.* (**A, B**) Scatterplots of the log_2_(average gene expression) during exposure to (**A**) cellobiose or (**B**) succinate vs. log_2_(fold change) in gene expression during exposure to (**A**) cellobiose or (**B**) succinate compared to carbon starvation in wild-type cells. (**C**) Scatterplot of the log_2_(fold change) in gene expression in wild-type cells exposed to cellobiose compared to carbon starvation vs. succinate compared to carbon starvation. Genes with a *p*_adj_ < 0.05 in succinate only are colored orange, a *p*_adj_ < 0.05 in cellobiose only are colored green, and a *p*_adj_ < 0.05 in both cellobiose and succinate are colored purple. Genes with a *p*_adj_ ≥ 0.05 in all comparisons are colored gray. (**D, E**) Scatterplots of the log_2_(average gene expression) during exposure to (**D**) cellobiose or (**E**) succinate vs. log_2_(fold change) in gene expression during exposure to (**D**) cellobiose or (**E**) succinate compared to carbon starvation in *cbr1*∆ cells. (**F, G**) Scatterplots of the log_2_(fold change) in gene expression in wild-type vs. *cbr1*∆ cells exposed to (**F**) cellobiose or (**G**) succinate compared to carbon starvation. Genes with a *p*_adj_ < 0.05 in wild-type cells only are colored blue, a *p*_adj_ < 0.05 in *cbr1*∆ only are colored gold, and a *p*_adj_ < 0.05 in both *cbr1*∆ and wild type are colored magenta. Genes with a *p*_adj_ ≥ 0.05 in all comparisons are colored gray. For (**A**), (**B**), (**D**), and (**E**), genes with a *p*_adj_ < 0.05 are colored red if they had higher expression in the indicated carbon source compared to carbon starvation and blue if they had lower expression in the indicated carbon source compared to carbon starvation. The five genes determined to be directly or indirectly activated by *CBR1* across multiple carbon sources (*CBR1* core regulon) are highlighted in orange and labeled with their respective protein IDs. FC stands for fold change and TPM stands for transcripts per million. All RNAseq experiments had three biological replicates.
(TIF)

S4 Fig Cbr1 directly or indirectly regulates a core regulon of five genes. Volcano plots of the log_2_(fold change) in gene expression versus the − log_10_(*p*_adj_) of the comparison in (**A** and **B**) wild-type cells exposed to (**A**) cellobiose vs. no carbon and (**B**) succinate vs. no carbon, (**C** and **D**) *cbr1*∆ cells exposed to (**C**) cellobiose vs. no carbon and (**D**) succinate vs. no carbon, and (**E–H**) *cbr1*Δ relative to wild-type cells when cells were exposed to (**E**) cellobiose, (**F**) succinate, (**G**) media lacking a carbon source, or (**H**) fucose. Genes with *p*_adj_ < 0.05 are indicated in red or blue if they had higher or lower expression in (**A–D**) cellobiose or succinate vs. no carbon or (**E–H**) *cbr1*Δ cells vs. wild-type cells, respectively. Genes differentially expressed by a least 4-fold in at least three of the four *cbr1*∆ vs. wild type comparisons (*CBR1* core regulon) are circled in orange. Genes in gray were not significantly differentially expressed in (**A–D**) cellobiose or succinate vs. no carbon or (**E–H**) *cbr1*Δ relative to wild-type cells. Black dotted lines indicate 4-fold differential expression and *p*_adj_ = 0.05. (**A–G**) Standard RNAseq was used to determine gene expression and fold change during exposure to cellobiose, succinate, and carbon starvation. (**H**) 3′ RNAseq was used to determine gene expression and fold change during exposure to fucose. Standard RNAseq and 3′ RNAseq data cannot be directly compared. The expression of *CBR1* was left out of the volcano plots involving its deletion strain. All RNAseq experiments had three biological replicates.
(TIF)

S5 Fig Cbr1 is present in the nucleus during exposure to glucose, cellobiose, succinate, and carbon starvation. (**A**) Expression (transcripts per million, TPM) of *CBR1* during exposure to media containing succinate, cellobiose, or lacking a carbon source (no carbon). Bars are the average and dots are the individual data points of three biological replicates. The data underlying **A** can be found in S2 Data. (**B**) Cells with the histone subunit Hta1 (H2A) tagged with mRuby2 (Hta1-mRuby2) and Cbr1 tagged with GFP (Cbr1-mEGFP) were exposed to the indicated carbon source for 4 h prior to imaging. Fluorescent microscopy was performed using a Nikon Eclipse Ti2-E inverted microscope equipped with a prime BSI express CMOS camera using a CFI Plan Apochromat 100× oil objective. The scale bar is 5 µm. Images are representative of three biological replicates.
(TIF)

S6 Fig Cbr1-dependent expression of secreted β-glucosidase genes *BGL1* (RTO4_16717) and *BGL2* (RTO4_16716) is necessary for cellobiose and gentiobiose but not TCA cycle intermediate or fucose utilization. (**A**) Growth curves (OD600) of wild-type, *cbr1*∆, *bgl1*∆ *bgl2*∆, and *bgl1*∆ *bgl2*∆ + *BGL1 BGL2* cells grown on YNB 8.5 mM citrate, YNB 1% succinate, or YNB 1% fucose. (**B**) Cell density (OD600) at the time of supernatant harvest (20 h) for cultures corresponding to Figs 3C and S6C β-glucosidase activity. (**C**) β-glucosidase activity of supernatants from *cbr1*∆, *bgl1*∆, and *bgl1*∆ *bgl2*∆ strains grown on cellobiose. β-glucosidase activity is presented as µM of p-nitrophenyl released from 5 mM p-nitrophenyl-β-D-glucopyranoside. Twice as much supernatant was used as input for these β-glucosidase activity assays than for all other β-glucosidase activity assays (see Materials and methods). (**D**) Cell density (OD600) at the time of supernatant harvest for cultures corresponding to Figs 3E, S6E, and S6F. Cultures were allowed to grow until they reached an OD600 of approximately 0.7. (**E**) β-glucosidase activity as measured by a p-nitrophenyl-β-D-glucopyranoside assay of culture supernatants used for the glucose release assay (Figs 3E and S6F). β-glucosidase activity is presented as µM of p-nitrophenyl released from 5 mM p-nitrophenyl-β-D-glucopyranoside. (**F**) Glucose released from 1% trehalose, 1% maltose, 1% lactose, or 1% sucrose after incubation with the indicated succinate culture supernatant. Culture supernatants were harvested when a culture reached an OD600 of 0.7. Growth (OD600) at time of supernatant harvest is in S6D Fig. (**A**) Lines are the average and bars are the standard deviation of three biological replicates. (**B–F**) Bars are the average and points are the individual data points of (**B** and **C**) five biological replicates for *cbr1*∆ cells and six biological replicates for all other strains or (**D–F**) three biological replicates. The data underlying this figure can be found in S2 Data. *p_adj_ < 0.05 and **p_adj_ < 10^−4^ as determined by (**A**) pairwise comparisons of estimated marginal means with a Holm multiple comparison correction or (**B–F**) one-way ANOVA with a TukeyHSD post-hoc test. Exact *p*-values are in S3 Data.
(TIF)

S7 Fig Secreted β-glucosidases in spent supernatants are sufficient to complement the growth of *cbr1∆* and *bgl1*∆ *bgl2*∆ cells on cellobiose. (**A**) Cell density (OD600) of wild-type and *cbr1∆* + *P*_*AAC1*_*-BGL1* cells grown in YNB 1% cellobiose at the time of the supernatant harvest (16 h) for cultures corresponding to the β-glucosidase activity in culture supernatants in S7B Fig. (**B**) β-glucosidase activity present in the culture supernatant of wild-type and *cbr1∆* + *P*_*AAC1*_*-BGL1* cells grown on YNB 1% cellobiose for 16 h. β-glucosidase activity is presented as µM of p-nitrophenyl released from 5 mM p-nitrophenyl-β-D-glucopyranoside. (**C**) β-glucosidase activity of culture supernatants of wild-type cells grown in YNB with 1% of the indicated carbon source. (Supernatants were used to supplement media in Figs 4D–K and S7D–S7G.) β-glucosidase activity is presented as µM of p-nitrophenyl released from 5 mM p-nitrophenyl-β-D-glucopyranoside. (**D**) Normalized growth (OD600) of the indicated strains in YNB 1% cellobiose supplemented with the indicated concentration of spent supernatant from wild-type (WT) cells grown in YNB 1% glucose. To account for growth due to nutrients remaining in the supplemented supernatant, growth was normalized by subtracting the growth of each replicate on media lacking a carbon source supplemented with the same concentration of spent supernatant. (**E–G**) Growth curves (OD600) of the indicated strains grown in YNB 1% cellobiose and YNB no carbon supplemented with the indicated concentration of culture supernatant from wild-type cells grown on glucose without normalization. (**A–C**) Bars are the average and points are the individual data points of three biological replicates. (**D–G**) Lines are the average and bars are the standard deviation of three biological replicates. The data underlying this figure can be found in S2 Data. (**A** and **B**) *p_adj_ < 0.05 as determined by Welch’s two-sample *t* test. (**C**) **p_adj_ < 10^−6^ as determined by a one-way ANOVA with a Tukey HSD post-hoc test. (**D**) Colored stars indicate a significant difference in growth of the respectively, colored strain compared to growth of that strain without supernatant supplementation. (**E–G**) Colored stars indicate a significant difference in growth of the respectively colored strain in YNB cellobiose + the indicated concentration of supernatant compared to YNB no carbon + the indicated concentration of supernatant. (**D–G**) *p_adj_ < 0.05 and **p_adj_ < 10^−3^ as determined by pairwise comparisons of estimated marginal means with a Holm multiple comparison correction. Exact *p*-values are in S3 Data.
(TIF)

S8 Fig Correlation between mRNAseq and 3′ RNAseq data. (**A**) *BGL1* expression relative to *ACT1* expression in wild-type cells exposed to cellobiose for the indicated amount of time. Bars are averages and dots are individual data points of three biological replicates. The data underlying **A** can be found in S2 Data. (**B, C**) Comparison of gene expression measured as DESeq2 normalized counts [91] in (**B**) wild-type or (**C**) *cbr1*∆ cells exposed to cellobiose for 8 h for mRNAseq or 4 h for 3′ RNAseq. Linear regression best-fit line and *R*^2^ values are displayed on each graph, *p* < 10^−15^.
(TIF)

S9 Fig The transcriptional profile of cells lacking *BGL1* and *BGL2* is more similar to the transcriptional profile of *cbr1*∆ cells than wild-type cells during exposure to cellobiose. (**A**) Hierarchical clustering using Euclidean distance of gene expression data of wild-type, *cbr1*∆, and *bgl1*∆ *bgl2*∆ cells exposed to cellobiose for 4 h for all genes with *p*_adj_ < 0.05 in at least one of the following pairwise comparisons: wild-type cells compared to *bgl1*∆ *bgl2*∆ cells, wild-type cells compared to *cbr1*∆ cells, or *bgl1*∆ *bgl2*∆ cells compared to *cbr1*∆ cells. (**B–D**) Volcano plots of the log_2_(fold change) in gene expression versus the −log_10_(*p*_adj_) of the comparison in (**B**) *bgl1*∆ *bgl2*∆ relative to wild-type cells exposed to cellobiose, (**C**) *cbr1*∆ relative to wild-type cells exposed to cellobiose, or (**D**) *bgl1*∆ *bgl2*∆ relative to *cbr1*∆ cells exposed to cellobiose as measured by 3′ RNAseq. Genes with *p*_adj_ < 0.05 are indicated in red or blue if they had higher or lower expression in (**B**) *bgl1*∆ *bgl2*∆ than wild-type cells, (**C**) *cbr1*Δ cells than wild-type cells, or (**D**) *bgl1*∆ *bgl2*∆ than *cbr1*∆ cells, respectively. Genes in the *CBR1* core regulon are circled in orange. Genes in gray were not significantly differentially expressed in (**B**) *bgl1*∆ *bgl2*∆ compared to wild-type cells, (**C**) *cbr1*Δ cells compared to wild-type cells, or (**D**) *bgl1*∆ *bgl2*∆ compared to *cbr1*∆ cells. Black dotted lines indicate 4-fold differential expression and *p*_adj_ = 0.05. The expression of *CBR1*, *BGL1*, and *BGL2* were left out of the volcano plots involving their respective deletion strains. All RNAseq experiments had three biological replicates.
(TIF)

S10 Fig Growth of *tct1*∆ (RTO4_13825∆), RTO4_10339∆, and RTO4_11229∆ cells on TCA cycle intermediates, cellobiose, and fucose. (**A, B**) Growth (OD600) of the indicated strains in YNB with 1% of the indicated carbon source for all carbon sources except citrate, which is at 8.5 mM. Lines are the average and bars or colored bands are the standard deviation of three biological replicates. The data underlying this figure can be found in S2 Data.
(TIF)

S11 Fig *TCT1* is required for citrate uptake. (**A**) Growth (OD600) of the indicated strains in YNB 8.5 mM citrate over the course of the citrate uptake assay in Fig 6B. (**B**) Citrate remaining in 8.5 mM citrate-phosphate buffer inoculated with the indicated strain at the indicated amount of time post-inoculation. Dotted black line indicates the measured initial citrate concentration in the citrate-phosphate buffer. (**C**) Growth (OD600) of the indicated strains in YNB 1% cellobiose, succinate, or fucose. Lines are the averages and (**A, B**) dots are the individual replicates or (**C**) bars are standard deviation of three biological replicates. The data underlying this figure can be found in S2 Data. *p_adj_ < 0.003 and **p_adj_ < 10^−3^ as determined by a two-way ANOVA with a TukeyHSD post-hoc test. Exact *p*-values are in S3 Data.
(TIF)

S12 Fig *TCT1* is not required to activate the transcriptional response to citrate. Volcano plots of the log_2_(fold change) in gene expression versus the −log_10_(*p*_adj_) of the comparison in (**A**) *cbr1*∆ relative to wild-type cells exposed to citrate, (**B**) *tct1*∆ relative to wild-type cells exposed to citrate, or (**C**) *cbr1*∆ relative to *tct1*∆ cells exposed to citrate as measured by 3′ RNAseq. Genes with *p*_adj_ < 0.05 are indicated in red or blue if they had higher or lower expression in (**A**) *cbr1*Δ cells than wild-type cells, (**B**) *tct1*∆ cells than wild-type cells, or (**C**) *cbr1*∆ cells than *tct1*∆ cells, respectively. Genes in the *CBR1* core regulon are circled in orange. Genes in gray were not significantly differentially expressed in **A**) *cbr1*Δ cells relative to wild-type cells, (**B**) *tct1*∆ cells relative to wild-type cells, or (**C**) *cbr1*∆ cells relative to *tct1*∆ cells. Black dotted lines indicate 4-fold differential expression and *p*_adj_ = 0.05. The expression of *CBR1* and *TCT1* were left out of the volcano plots involving their respective deletion strains. All RNAseq experiments had three biological replicates.
(TIF)

S13 Fig Cbr1 directly or indirectly inhibits carbon catabolite repression during disaccharide and proline utilization. Area under the curve calculations for growth curves (OD600) of the indicated strains on the indicated carbon sources with the indicated concentration of 2-deoxyglucose (2-DG) or allyl alcohol (AA) from (**A, B**) Fig 7B, (**C**) Fig 7E, (**D**) Fig 7G, and (**E**) Fig 7H. Bars are the average and dots are individual replicates of 3 biological replicates. The data underlying this figure can be found in S2 Data. *p_adj_ < 10^−3^ and **p_adj_ < 10^−8^ as determined by two-way ANOVA with a TukeyHSD post-hoc test. Exact *p*-values are in S3 Data.
(TIF)

S14 Fig *AGL1* (RTO4_9135) is required for maltose and sucrose utilization. (**A** and **C**) (**A**) *AGL1* expression or (**C**) RTO4_14602 expression relative to *ACT1* expression in wild-type and *cbr1*∆ cells exposed to the indicated carbon sources for 8 h, unless indicated otherwise. Bars are the averages and dots are individual data points of three biological replicates. Letters signify statistically different groups (*p*_adj_ < 0.05) as determined by a two-way ANOVA with a TukeyHSD post-hoc test. (**B**) Growth (OD600) of the indicated strains in YNB with 1% of the indicated carbon source. Lines are the average and colored bands are the standard deviation of three biological replicates. *p_adj_ = 0 as determined by pairwise comparisons of estimated marginal means with a Holm multiple comparison correction. Exact *p*-values are in S3 Data. The data underlying this figure can be found in S2 Data.
(TIF)

S15 Fig Deletions of genes in the *CBR1* core regulon do not result in a growth phenotype when 2-deoxyglucose is added to media containing nonpreferred carbon sources. Growth (OD600) of the indicated strains in YNB with 1% of the indicated carbon source supplemented with the indicated concentration of 2-deoxyglucose. Lines are the average and bars are the standard deviation of three biological replicates. The data underlying this figure can be found in S2 Data.
(TIF)

S16 Fig *N. crassa* CLR-2 does not regulate carbon catabolite repression in *N. crassa.* Mycelial dry weights of wild-type and ∆*clr-2 N. crassa* cells directly inoculated into 100 mL VMM NH_4_Cl containing 1% (**A**) trehalose or (**B**) xylose supplemented with the indicated concentration of 2-deoxyglucose and grown for 24 h prior to harvesting. Bars are the averages and dots are the individual data points of three biological replicates. The data underlying this figure can be found in S2 Data.
(TIF)

S1 Table *R. toruloides* strains used in this study. *Hyg^*R*^ stands for hygromycin resistance cassette. **Nat^*R*^ stands for nourseothricin resistance cassette. ***G418^*R*^ stands for G418 resistance cassette. ****RTO4_16716 and RTO4_16717 are adjacent in the genome, so the *bgl1*Δ *bgl2*Δ strain was generated via single transformation of the nourseothricin resistance cassette to remove both open reading frames from the genome, and a genomic fragment containing both genes was used to complement the deletion of *BGL1* and *BGL2* in the *bgl1∆ bgl2∆ + BGL1 BGL2* strain.
(DOCX)

S2 Table *N. crassa* strains used in this study. *FGSC stands for Fungal Genetics Stock Center [83].
(DOCX)

S3 Table Carbon sources used in this study. (DOCX)

S4 Table RNA sequencing method. (DOCX)

S5 Table RT-qPCR primers used in this study. (DOCX)

S1 Data Tree file of the phylogenetic tree in Fig 1A. (TXT)

S2 Data Numerical values to generate all bar and line graphs. (XLSX)

S3 Data Numbers of replicates for all experiments and exact p-values for all comparisons. (XLSX)

S4 Data Expression of all genes during exposure of wild-type and *cbr1∆* cells to cellobiose, succinate, and media lacking a carbon source for 8 h as measured by standard RNAseq and wild-type and *cbr1∆* cells exposed to fucose for 8 h as measured by 3′ RNAseq. (XLSX)

S5 Data Differential gene expression calculated by DESeq2 [91] in the indicated comparisons as measured by standard RNAseq of wild-type and *cbr1∆* cells exposed to cellobiose, succinate, and media lacking a carbon source for 8 h or 3′ RNAseq of wild-type and *cbr1∆* cells exposed to fucose for 8 h. (XLSX)

S6 Data *R. toruloides* GFF3 file made using peaks2utr [94] with improved 3′ UTR annotations generated using the 3′ RNAseq data of wild-type cells exposed to: YNB 1% fucose, YNB 1% cellobiose, YNB 8.5 mM citrate, YNB 1% succinate, and VMM NH_4_Cl 2% glucose. We subsequently used this GFF3 file to analyze all 3′ RNAseq data.
(TXT)

S7 Data Expression of all genes during exposure of wild-type, *cbr1*∆, and *bgl1*∆ *bgl2*∆ cells to cellobiose for 4 h as measured by 3′ RNAseq. (XLSX)

S8 Data Differential gene expression calculated by DESeq2 [91] in the indicated comparisons as measured by 3′ RNAseq of wild-type, *cbr1∆*, and *bgl1∆ bgl2∆* cells exposed to cellobiose for 4 h. (XLSX)

S9 Data Expression of all genes during exposure of wild-type, *cbr1*∆, and *tct1*∆ cells to citrate for 24 h as measured by 3′ RNAseq. (XLSX)

S10 Data Differential gene expression calculated by DESeq2 [91] in the indicated comparisons as measured by 3′ RNAseq of wild type, *cbr1∆*, and *tct1∆* cells exposed to citrate for 24 h. (XLSX)

S11 Data KEGG orthology IDs for genes in the *R. toruloides* genome assigned by BlastKOALA [99]. (TXT)

S12 Data Tree file of the phylogenetic tree in S1 Fig. (TXT)

## Abbreviations

2-DG: 2-deoxyglucose
AA: allyl alcohol
BLASTP: Basic Local Alignment Search Tool for proteins
CPM: Counts per million
FGSC: Fungal Genetics Stock Center
FUDR: 5-fluorodeoxyuridine
GEO: Gene Expression Omnibus
GO: Gene Ontology
KEGG: Kyoto Encyclopedia of Genes and Genomes
NCBI: National Center for Biotechnology Information
RNAseq: RNA sequencing
RT-qPCR: reverse transcription quantitative PCR
TCA: tricarboxylic acid
UTRs: untranslated regions
VMM: Vogel’s minimal medium
WT: wild type
YNB: yeast nitrogen base
YPD: yeast peptone dextrose
