# Supplementary material for: The carbohydrate utilization regulator Cbr1 coordinates nutrient-specific gene activation with selective carbon catabolite repression in a basidiomycete yeast
Source: PLoS Biol. 2026 Sep 1;24(9):e3003983. doi: 10.1371/journal.pbio.3003983 (PMC13561427; doi:10.1371/journal.pbio.3003983)
Supplement: Supplementary file 1 [file pbio.3003983.s017.docx]

| **Strain name** | **Genotype** | **Source** |
| --- | --- | --- |
| Wild type | *yku70*(RTO4_11920)Δ*::Hyg^R^** | [80] |
| *cbr1*Δ | *cbr1*(RTO4_13588)∆*::Nat^R^** yku70*Δ*::Hyg^R^* | This study |
| *cbr1∆ + CBR1* | *cbr1*Δ*::Nat^R^ yku70∆::CBR1:G418^R^**** | This study |
| *HTA1-mRuby2 CBR1-mEGFP* | *HTA1*(RTO4_12090)*-mRuby2 CBR1-mEGFP:Nat^R^ yku70*Δ*::Hyg^R^* | This study |
| RTO4_10339*∆* | RTO4_10339*∆::G418^R^* *yku70*Δ*::Hyg^R^* | This study |
| RTO4_11229*∆* | RTO4_11229*∆::G418^R^* *yku70*Δ*::Hyg^R^* | This study |
| *tct1∆* | *tct1*(RTO4_13825)*∆::G418^R^* *yku70*Δ*::Hyg^R^* | This study |
| *tct1∆ + TCT1* | *tct1∆::G418^R^* *yku70*Δ*::TCT1:Nat^R^* | This study |
| *bgl1∆* | *bgl1*(RTO4_16717)*∆::Nat^R^*  *yku70*Δ*::Hyg^R^* | This study |
| *bgl1∆ + BGL1* | *bgl1∆::Nat^R^*  *yku70*Δ*::BGL1:G418^R^* | This study |
| *bgl2∆* | *bgl2*(RTO4_16716)*∆::Nat^R^*  *yku70*Δ*::Hyg^R^* | This study |
| *bgl1∆ bgl2∆* | *bgl1∆ bgl2∆*::*Nat^R^**** yku70*Δ*::Hyg^R^* | This study |
| *bgl1∆ bgl2∆* + *BGL1 BGL2* | *bgl1*∆ *bgl2*∆*::Nat^R^**** yku70∆::BGL1 BGL2:G418^R^***** | This study |
| *cbr1∆ + P_AAC1_-BGL1* | *cbr1∆::NatR*  *yku70*Δ*::P_AAC1_-BGL1:G418^R^* | This study |
| *tct1*∆ + *P_AAC1_-mRuby-TCT1* (1) | *tct1∆::G418^R^ yku70∆::P_AAC1_-mRuby2-TCT1:Nat^R^* (Transformant 1) | This study |
| *tct1*∆ + *P_AAC1_-mRuby-TCT1* (2) | *tct1∆::G418^R^ yku70∆::P_AAC1_-mRuby2-TCT1:Nat^R^* (Transformant 2) | This study |
| *agl1*∆ (1) | *agl1*(RTO4_9135)*∆::G418^R^* *yku70::Hyg^R^* (Transformant 1) | This study |
| *agl1*∆ (2) | *agl1∆::G418^R^* *yku70::Hyg^R^* (Transformant 2) | This study |
