# Supplementary material for: The carbohydrate utilization regulator Cbr1 coordinates nutrient-specific gene activation with selective carbon catabolite repression in a basidiomycete yeast
Source: PLoS Biol. 2026 Sep 1;24(9):e3003983. doi: 10.1371/journal.pbio.3003983 (PMC13561427; doi:10.1371/journal.pbio.3003983)
Supplement: Supplementary file 2 [file pbio.3003983.s018.docx]

| **Strain name** | **Genotype** | **Source** |
| --- | --- | --- |
| Wild type | Wild type *mat A* | FGSC* 2489 [83] |
| Δ*clr-2* | Δ*clr-2* (NCU08042)*::Hyg^R^ mat A* | FGSC 15834 [82] |
