# Supplementary material for: The carbohydrate utilization regulator Cbr1 coordinates nutrient-specific gene activation with selective carbon catabolite repression in a basidiomycete yeast
Source: PLoS Biol. 2026 Sep 1;24(9):e3003983. doi: 10.1371/journal.pbio.3003983 (PMC13561427; doi:10.1371/journal.pbio.3003983)
Supplement: Supplementary file 3 [file pbio.3003983.s019.docx]

| **Carbon source** | **Company** |
| --- | --- |
| 2-deoxyglucose | TCI |
| Acetate | EMD |
| Alanine | BeanTown Chemical |
| Arabinose | Chem-Impex |
| Cellobiose | RPI |
| Citrate | VWR |
| Coumaric acid | TCI |
| Ferulic acid | Ambeed |
| Fructose | VWR |
| Fucose | TCI |
| Fumaric acid | BeanTown Chemical |
| Galactose | BeanTown Chemical |
| Galacturonic acid | Thermo |
| Gentiobiose | Thermo |
| Glucose | VWR |
| Glutamate | Sigma |
| Glycine | EM Science |
| Malic acid | TCI |
| Maltose | BeanTown Chemical |
| Mannose | BeanTown Chemical |
| Proline | BeanTown Chemical |
| Succinic acid | VWR |
| Sucrose | TCI |
| Trehalose | TCI |
| Tween-80 (Polysorbate 80) | VWR |
| Xylose | Thermo |
| α-Ketoglutarate | Alfa Aesar |
