# Supplementary material for: The carbohydrate utilization regulator Cbr1 coordinates nutrient-specific gene activation with selective carbon catabolite repression in a basidiomycete yeast
Source: PLoS Biol. 2026 Sep 1;24(9):e3003983. doi: 10.1371/journal.pbio.3003983 (PMC13561427; doi:10.1371/journal.pbio.3003983)
Supplement: Supplementary file 4 [file pbio.3003983.s020.docx]

| **Media** | **Strains** | **Hours exposed to carbon source** | **RNA sequencing method** | **Relevant figure** |
| --- | --- | --- | --- | --- |
| YNB 1% cellobiose,  YNB 1% succinate, and YNB no carbon | Wildtype and *cbr1∆* | 8 h | Whole transcript mRNA sequencing | Fig 2A-2C, 2E, S3, S4A-S4G, S5A, S8, and Dataset S4 and S5 |
| YNB 1% fucose | Wildtype and *cbr1∆* | 8 h | 3’ mRNA sequencing | Fig 2D, 2E, S4H, and Dataset S4 and S5 |
| YNB 1% cellobiose | Wildtype, *cbr1∆,* and *bgl1∆ bgl2∆* | 4 h | 3’ mRNA sequencing | Fig 5A-5C, S8, S9, and Dataset S7 and S8 |
| YNB 8.5 mM citrate | Wildtype, *cbr1∆,* and *tct1∆* | 24 h | 3’ mRNA sequencing | Fig 6E, S12, and Dataset S9 and S10 |
| YNB 1% succinate | Wild type | 24 h | 3’ mRNA sequencing | Only used to annotate 3’ UTRs |
| VMM NH_4_Cl 2% glucose | Wild type | 8 h | 3’ mRNA sequencing | Only used to annotate 3’ UTRs |
