# Supplementary material for: The carbohydrate utilization regulator Cbr1 coordinates nutrient-specific gene activation with selective carbon catabolite repression in a basidiomycete yeast
Source: PLoS Biol. 2026 Sep 1;24(9):e3003983. doi: 10.1371/journal.pbio.3003983 (PMC13561427; doi:10.1371/journal.pbio.3003983)
Supplement: Supplementary file 5 [file pbio.3003983.s021.docx]

| **Gene** | **Forward 5’ 🡪 3’** | **Reverse 5’ 🡪 3’** |
| --- | --- | --- |
| *ACT1* | CGTCCTCTCGCTCTATGCCTC | CTTGATGAGGTAGTCGGTCAGGTC |
| *BGL1* | CGGGTGGAATCATGTGCTCGTA | CGTACCAGTCCGTGACGACAAAG |
| *AGL1* | AGGTTCGACGGGCCATCTAC | GGGAAGTCGAGGTACTTGCTGTA |
| RTO4_9774 | CTCGAACTTCTCGCCTGAGATGGT | GGTGACGCTCGATCTGGTTGAC |
| RTO4_14602 | CGCAATCTCGGAACAGTCGAT | AGGTGTGGTCCATGCCAAAG |
